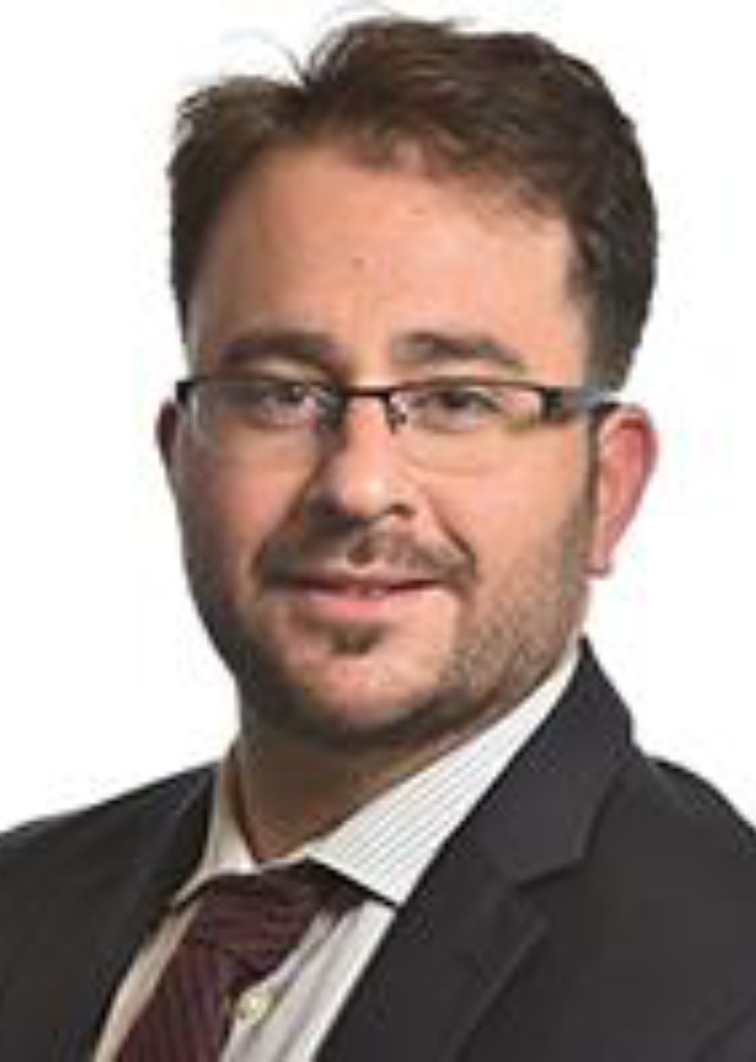# Progress on Photo‐, Electro‐, and Photoelectro‐Catalytic Conversion of Recalcitrant Polyethylene, Polypropylene, and Polystyrene ‐ A Review

**DOI:** 10.1002/cssc.202401714

**Published:** 2024-12-05

**Authors:** Bhanupriya Boruah, Juan A. Lopez‐Ruiz

**Affiliations:** ^1^ Institute for Integrated Catalysis Pacific Northwest National Laboratory WSU-PNNL Bioproducts Institute 902 Battelle Blvd Richland WA 99352 USA

**Keywords:** Plastic upcycling, photocatalysis, electrocatalysis, photoelectrocatalysis

## Abstract

Recalcitrant waste plastics such a polyethylene, polypropylene, and polystyrene are difficult to recycle and are mostly disposed of in landfills and eventually leached into the environmental as micro‐ and nano‐plastics. This review explores how photo‐, electro‐, and combined photoelectro‐catalytic processes can assist in the degradation and upcycling of waste plastic into different chemicals and mitigate their release to the environment. In this work, we discuss how the different reaction mechanisms proceed, explore the current relevant literature, and highlight the developments needed to advance the field.

## Graphical abstract

Recalcitrant waste plastic can be degraded into fuels and chemicals using innovative methods like photo‐, electro‐, and photoelectro‐catalytic processes. This review highlights advancements in converting polyethylene, polypropylene, and polystyrene into valuable chemicals, unlocking new potential for sustainable upcycling.

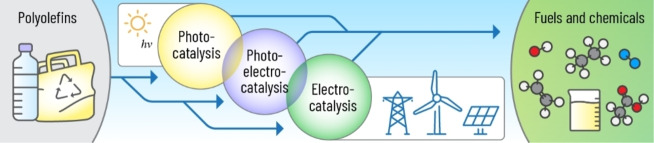

## 1 Introduction

**Figure 1:**
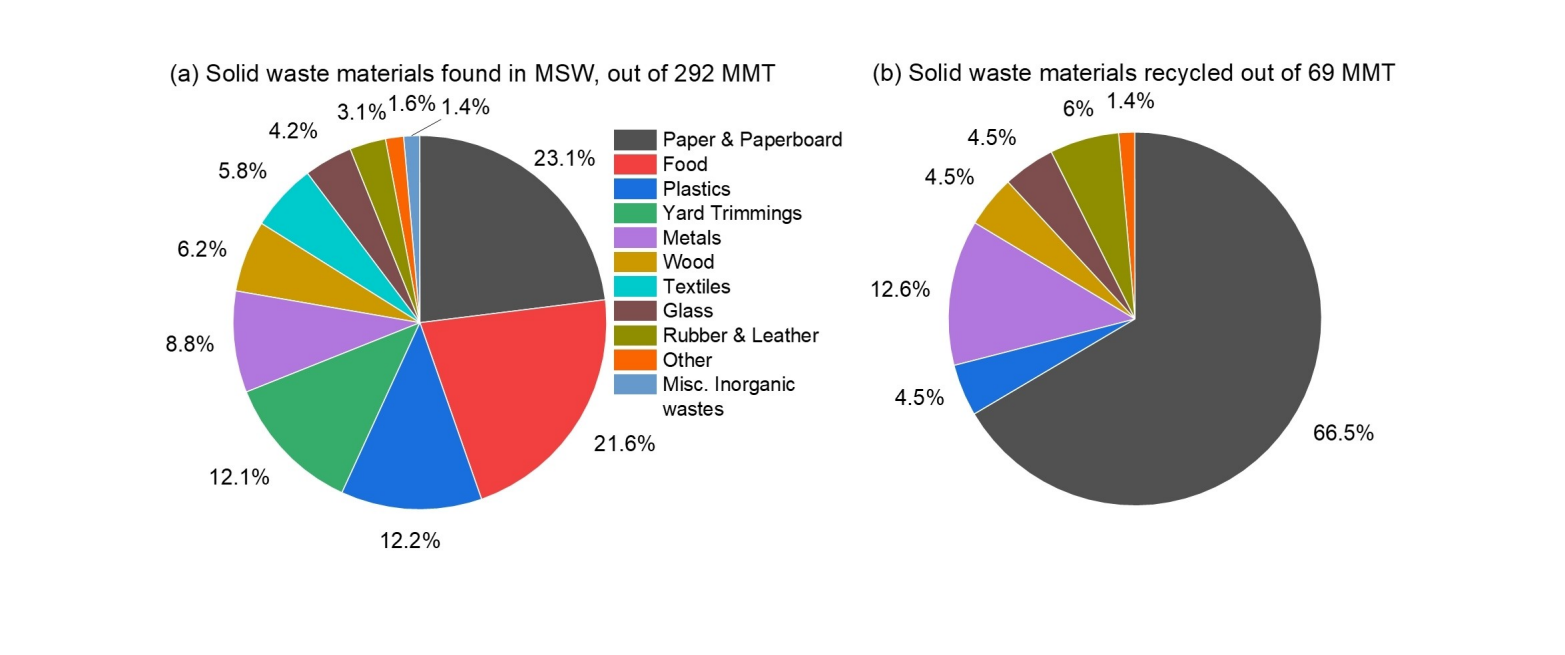
Breakdown of solid waste materials (a) found and (b) recycled in municipal solid waste. The U.S. generated 292 MMT of solid waste in 2018 but only 69 MMT was recycled.

Plastic‐derived products are vital to human activity due to their low cost and stability. For these reasons, their recycling has been mostly ignored (i. e., it is not cost effective to recycle because pristine is low cost) and polymers are mostly disposed of in landfills.[^1^, ^2^] According to a U.S. Environment Protection Agency report on municipal solid waste (MSW) generation in 2018, out of 292 million metric tons (MMT) of waste, plastics contributed about 12.2 % (35.7 MMT). Only 69 MMT of the total MSW was recycled (23.6 %), of which plastic recycling comprised 4.5 % (3.09 MMT), as shown in Figure 1.^[3]^

**Figure 2:**
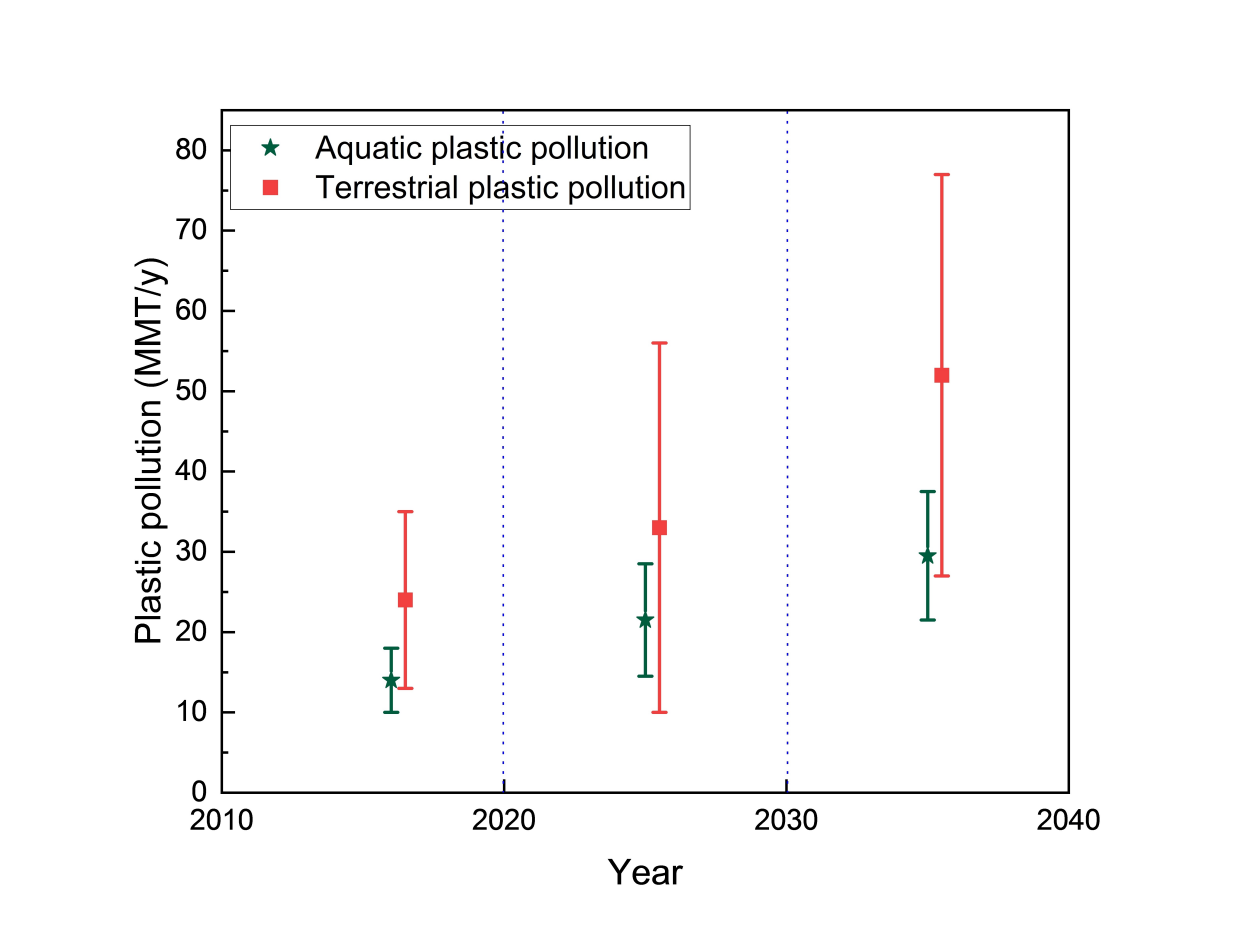
Rates of plastic pollution entering aquatic and terrestrial systems from Monte Carlo simulations based on business‐as‐usual scenario.

On average, global release of plastic waste into the aquatic ecosystem ranges from 9 to 15 MMT per year (MMT/y), and 13 to 25 MMT/y is released into the terrestrial environment.[^4^, ^5^] If plastic recycling does not improve, the amount of plastic released globally in 2016 is expected to more than double by 2040 (Figure 2).[^4^, ^5^] Furthermore, the amount of global life‐cycle greenhouse gas emissions of conventional plastics was 1.7 gigatons (GT) CO_2_ in 2015, which is predicted to nearly quadruple by 2050 (to 6.5 GT of CO_2_) under the current trajectory due to increased demand for plastic and lack of efficient plastic recycling infrastructure.^[6]^

The lack of action on plastic recycling has resulted in the contamination of the food and water supplies, mainly as micro‐ and nano‐plastics that are even more difficult to remove as they are stable and diluted, representing an environmental threat. For example, multiple studies have shown that animals and plants can ingest accumulated micro‐ and nano‐plastics in plants.[^7^, ^8^] Some studies show these ingested plastics may lead to cancer, lung disease, and reproductive toxicity.[^9^, ^10^, ^11^] Therefore, effective technologies for the treatment and recycling of plastic wastes should be developed to alleviate the negative impacts on the natural environment.

**Figure 3:**
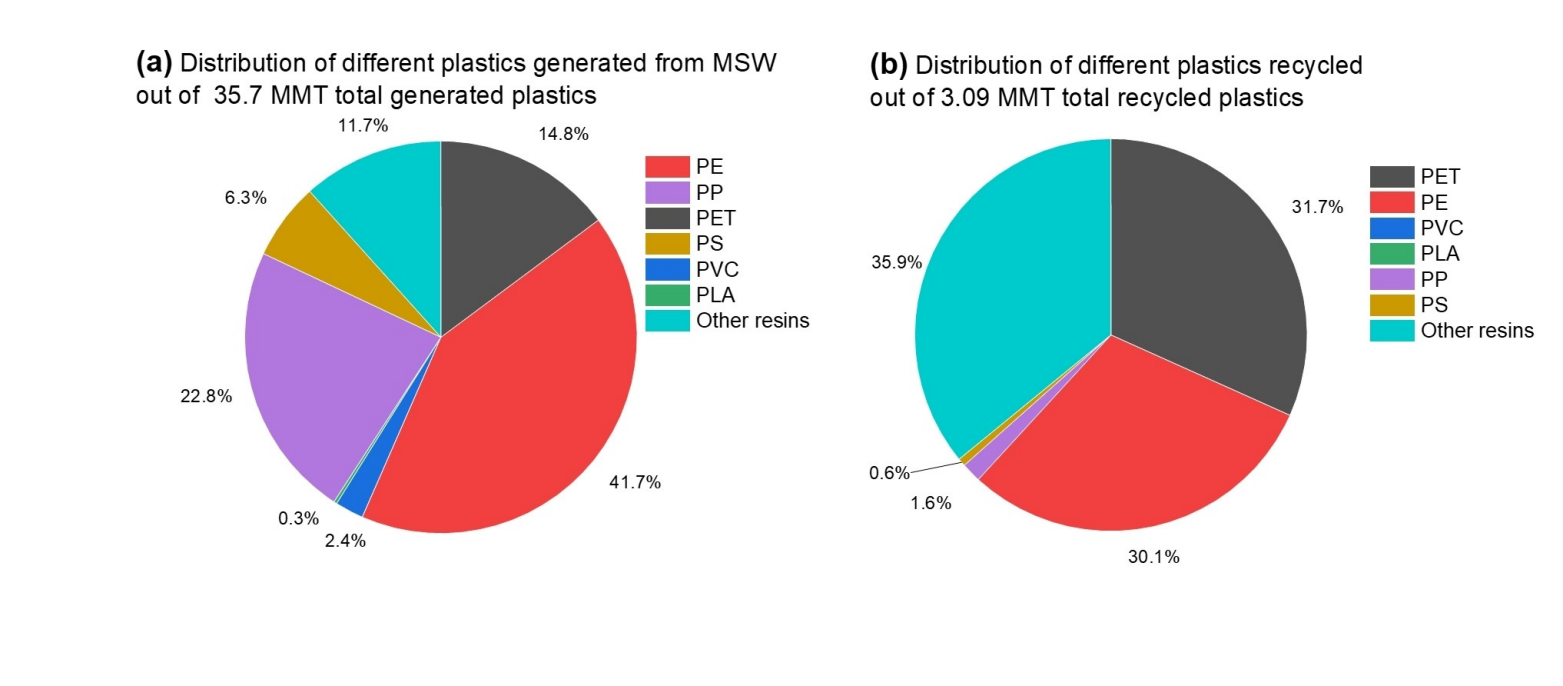
Breakdown of plastics (a) found and (b) recycled in municipal solid waste. The U.S. generated 35.7 MMT of waste plastic in 2018 but only 3.09 MMT was recycled. Here, PE represents polyethylene, PP represents polypropylene, PET represents polyethylene terephthalate, PS represents polystyrene, PVC represents polyvinyl chloride, PLA represents polylactic acid, and others represent diverse resins such as polycarbonates, acrylic, nylon, fiberglass.

In the plastic market, polyolefins account for 40 % to 50 % of global plastic production and waste.[^12^, ^13^] From a study on distribution of various plastics generated from MSW (Figure 3), out of 35.7 MMT total plastics generation, polyethylene (PE) is the largest contributor at ≈42 % (14.8 MMT).^[3]^ Polypropylene (PP) follows at ≈23 % (8.15 MMT). Polystyrene (PS) contributes ≈6.3 % (2.2 MMT). Of the total 3.09 MMT of recycled plastics, PE recycling accounts for 31.7 %, PP for 1.6 %, and PS for 0.6 %.^[3]^ Everyday items made from PE include milk jugs, hygiene bottles, and bubble wrap.[^14^, ^15^, ^16^] PP‐based products include bottle caps, straws, and yogurt containers.[^17^, ^18^] PS‐based products include take‐away containers and foam cups.^[19]^

PE and PP are crystalline in nature and linked by carbon‐carbon (C‐C) and carbon‐hydrogen (C‐H) bonds, which makes them chemically unreactive and highly resistant to decomposition.[^20^, ^21^, ^22^] PS has an aromatic ring that increases its rigidity and contributes to its amorphous form.^[23]^ The high stability of these polymers can be correlated to the high bond dissociation enthalpy (BDE) of the C‐C bond. For example, the BDE main‐chain C‐C bond is 364.3 kJ/mol for PE and 357.1 kJ/mol for PP. The branched‐chain C‐CH_3_ bond in PP is 361.9 kJ/mol. Similarly, the BDE of the PS C‐C bond is 331.5 kJ/mol, while the branched‐chain C‐C (aromatic) bond BDE is 424.1 kJ/mol.^[24]^ Polyvinyl chloride (PVC) has a C‐C bond BDE of 373.8 kJ/mol, while the branched‐chain C‐Cl bond BDE is 355.6 kJ/mol. Polyethylene terephthalate (PET) has a similar C‐C bond BDE at 358.2 kJ/mol and an oxygen‐carbon (O‐C) bond BDE of 381.4 kJ/mol. On the other hand, bisphenol A polycarbonate has a C‐CH bond BDE of 302.4 kJ/mol, while the O‐C carbonyl bond BDE is 332.8 kJ/mol. The BDE (based on the respective weakest bonds) is highest for PE, followed by PP, which might illustrate their chemical inertness. Additionally, the thermal stability order was reported to be PVC<PS<PP<PE.^[24]^ This inherent stability of plastics PE, PP, and PS poses challenges for recycling and environmental management, necessitating advanced and sustainable processing methods.

Currently, these plastics are upcycled using thermochemical routes such as pyrolysis and gasification at temperatures ranging from 500 °C to 1000 °C.[^25^, ^26^, ^27^] These processes convert plastic waste into a complex mixture of fuel‐like hydrocarbons, which require further processing, including separation, purification, and upgrading into raw polymer or fuel precursors.^[28]^ Additionally, the high temperatures, along with the use of molecular hydrogen (H_2_) or other fossil‐fuel‐derived reagents, increase the greenhouse gas emissions associated with thermal recycling. Therefore, renewable and sustainable approaches are urgently needed to mitigate the environmental impact of plastic waste.

**Figure 4:**
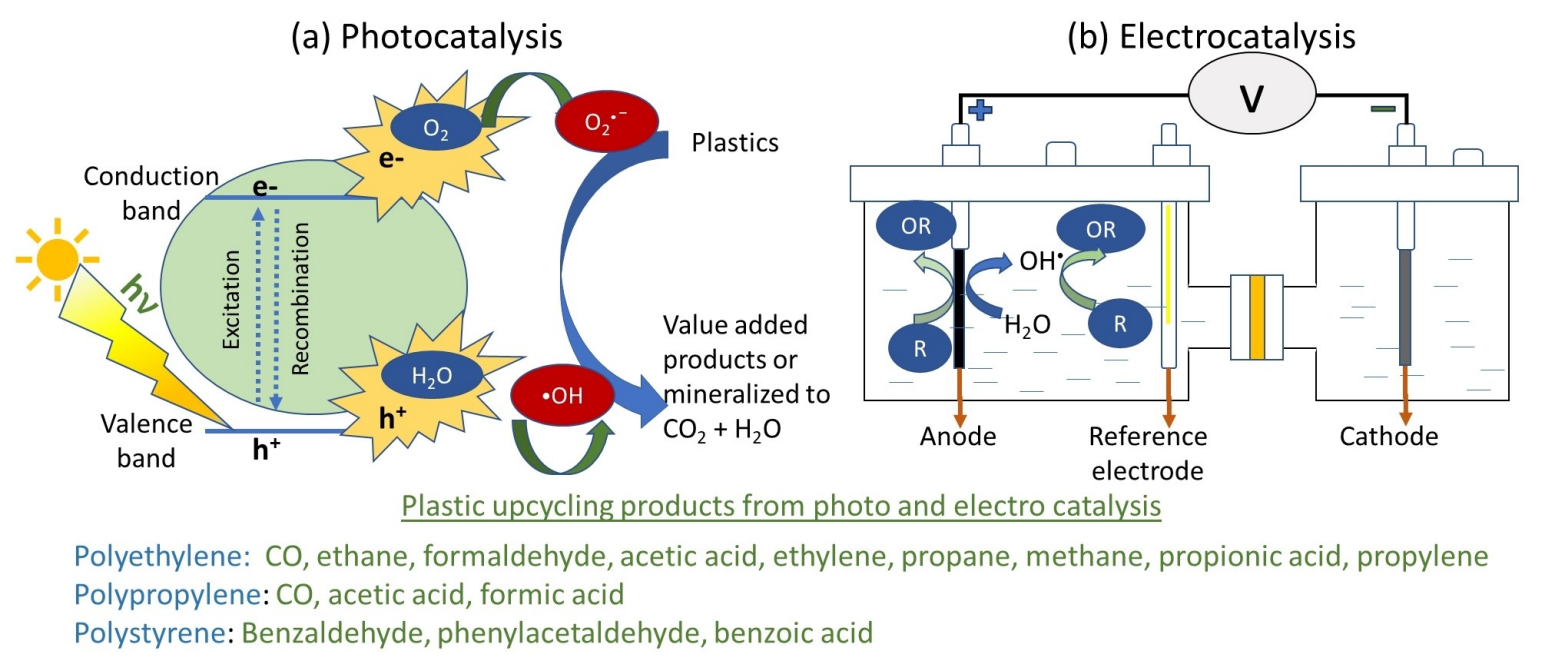
Schematic of photocatalysis and electrocatalysis for plastic conversion.

Photo‐ and electro‐catalytic processes are considered sustainable alternatives for plastic decomposition as they can be carried out at near atmospheric reaction conditions and would use renewable energy to drive the decomposition reaction. Photocatalysis (PCAT) uses light directly to drive the (photochemical) reaction; however, electrocatalysis (ECAT) uses electrical power to drive the electrochemical reaction. Both technologies can be used to depolymerize plastics. Figure 4 shows a schematic of polymer upcycling to value‐added products via PCAT and ECAT.

PCAT is a process that uses (1) incident light radiation as an energy source and (2) a catalytic material to accelerate chemical reactions.^[29]^ Semiconductor photocatalytic systems have garnered significant attention since the 1970s for various applications, including carbon dioxide (CO_2_) reduction, water splitting, and wastewater treatment.[^30^, ^31^, ^32^, ^33^] In the context of plastic treatment, photocatalysts such as TiO_2_ have shown promise due to their ability to generate reactive oxygen species (ROS) like superoxide radicals (O_2_^.−^) and hydroxyl radicals (⋅OH) upon light irradiation.[^34^, ^35^] These radicals play critical roles in breaking down complex plastic polymers into simpler, reusable materials. When TiO_2_ photocatalysts are irradiated with light of sufficient energy, hν (greater than or equal to its band gap energy of 3.2 eV), they produce charge carriers which are electrons (e^−^) and holes (h^+^). This excitation shifts e^−^ from the valence band (VB) to the conduction band (CB), creating h^+^ in the VB (Equation (1)). The excited e^−^ can reduce O_2_ to form O_2_^.−^ radicals while h^+^ react with H_2_O to generate ⋅OH **(**Equations (2) and (3)). Both O_2_^.−^ and ⋅OH radicals are highly reactive and can effectively oxidize plastics and other organic contaminants[^34^, ^36^, ^37^, ^38^, ^39^, ^40^, ^41^, ^42^] (Equation (4)). However, the efficiency of this process can be limited by the recombination of e^−^ and h^+^, decreasingg the availability of reactive species (Equation (5)).[^43^, ^44^] The generated ⋅OH radicals could break polymer chains and generate carbon‐centered radicals, Equation (6). These carbon‐centered radicals then drive further reactions (Equations (7) to (10)), resulting in polymer cleavage, incorporation of oxygen into polymer chains, and the formation of carbonyl groups. The decomposition of hydroperoxides (ROOH) to alkoxy radicals (RO⋅) and ⋅OH (Equations (8) and (9)) is the rate‐determining step in these reactions, initiating further degradation of polymer chains. Additionally, ketone photolysis contributes to polymer degradation through free‐radical generation (Equation (11)) and chain scission (Equation (12)). Ketones incorporated into the polymer backbone absorb photons, breaking C‐C bonds and leading to polymer scission under ultraviolet (UV) light. Further oxidation can completely mineralize the carbonyl intermediates into CO_2_ and water (Equation (13)).^[45]^ Thus, researchers have been developing modified photocatalysts that minimize recombination by doping the semiconductor with suitable metals or non‐metals and creating heterojunctions to improve charge separation and enhance photocatalytic efficiency.[^41^, ^46^, ^47^, ^48^, ^49^]

While much research has focused on the application of PCAT systems for water decontamination, there is a growing need to explore their potential for plastic degradation.[^50^, ^51^] By directing the research community′s attention toward optimizing PCAT systems specifically for plastic treatment, we can develop innovative solutions to transform plastic waste into valuable materials, contributing to environmental sustainability and resource efficiency.

(1) $${TiO}_{2}\;+\;h\nu \to {TiO}_{2}\;\left(e^{-}\;+\;h^{+}\right)$$

(2) $${\mathrm{TiO}}_{2}\;\left(e^{-}\right)+\;O_{2}\to O_{2}^{\cdot -}$$

(3) $$H_{2}O_{ads}\;+\;h^{+}\to \;\cdot \mathrm{OH}\;+\;H^{+}$$

(4) $$\mathrm{OH}\;+\;\mathrm{organic}\;\mathrm{compounds}/\mathrm{plastics}\to \;H_{2}O\;+\;{\mathrm{CO}}_{2}\;$$

(5) $${TiO}_{2}\;\left(e^{-}\right)\;+\;{TiO}_{2}\;\left(h^{+}\right)\;\to heat$$

(6) $$\left(-{\mathrm{CH}}_{2}-{\mathrm{CH}}_{2}-\right)n\;+\;\cdot \mathrm{OH}\to \left(-{\mathrm{CH}}_{2}-\cdot \mathrm{CH}-\right)n\;+\;H_{2}O\;$$

(7) $$\left(-{\mathrm{CH}}_{2}-\cdot \mathrm{CH}-\right)n\;+\;O_{2}\to \;\left(-\mathrm{HCOO}\cdot -{\mathrm{CH}}_{2}\right)n$$

(8) $$\left(-HCOO\cdot -CH_{2}-\right)n+\left(-CH_{2}-CH_{2}-\right)n\to \left(-HCOOH-CH_{2}-\right)n+\left(-CH_{2}-\cdot CH-\right)n$$

(9) $$\left(-\mathrm{HCOOH}-{\mathrm{CH}}_{2}-\right)n+h\nu \to \;\left(-\mathrm{HCO}\cdot -{\mathrm{CH}}_{2}-\right)n\;+\;\cdot \mathrm{OH}$$

(10) $$\left(-\mathrm{HCO}\cdot -{\mathrm{CH}}_{2}-\right)n\;+\;O_{2}\;+\;{\mathrm{TiO}}_{2}+h\nu \to \;\mathrm{Carbonyl}\;\mathrm{groups}$$

(11) $$\left(-{\mathrm{CH}}_{2}-\mathrm{CO}-{\mathrm{CH}}_{2}-\right)n+h\nu \to \left(-{\mathrm{CH}}_{2}-\mathrm{CO}\cdot \right)n+\left({\cdot \mathrm{CH}}_{2}-\right)n$$

(12) $$\left(-CH_{2}-CO-CH_{2}-\right)n+h\nu \to \left(-CH_{2}-CO\right)n+\left(\cdot CH_{2}=CH\right)n$$

(13) $$\left(-CH_{2}-HCO\right)\;+\;\left(-CH_{2}-COOH\right)+\;\left(-CH_{2}-CO-CH_{2}-\right)+\;O_{2}\;+\;TiO_{2}+h\nu \to \;+CO_{2}\;+\;H_{2}O$$

ECAT uses electrodes and a potential bias across an anode (oxidation) and a cathode (reduction) submerged in a suitable electrolyte solution to drive electrocatalytic reaction.^[52]^ The electrodes are made of active, conductive materials such as base‐ and noble‐group metals supported by chemically inert but conductive substrates (e. g., Ti, carbon, stainless steel). Cathode reactions focus on electrochemical hydrogenation and H_2_ evolution reactions. However, anode reactions can be categorized into (1) direct electrochemical oxidation, (2) indirect electrochemical oxidation, and (3) chemical depolymerization followed by electrochemical upcycling. In direct oxidation, the organic compound adsorbs onto the anode surface, loses e^−^ and protons (H^+^), or gains oxygen groups (O^−^), eventually fully oxidizing into CO_2_ and H_2_O. However, indirect oxidation refers to a process where the organic compounds are decomposed in the bulk solution by strong oxidants (e. g., O_2_⋅^−^, ⋅OH) or chlorine radicals (Cl⋅ and Cl_2_⋅^−^) generated at the electrode surface.^[53]^ Because the waste plastics are solids and insoluble in aqueous electrolytes, both direct and indirect ECAT processes are a challenging strategy for decomposing waste plastic. Alternatively, plastics can first undergo hydrolysis under strong acid or alkaline conditions^[54]^ followed by direct or indirect ECAT to fully degrade into CO_2_ and H_2_ or upcycle into value‐added products.[^55^, ^56^] ECAT represents a clean, sustainable technology, particularly when powered by renewable electricity. Unlike PCAT, which relies directly on solar energy, ECAT can use any form of electrical energy, facilitating its integration into the renewable energy grid. Additionally, ECAT allows for the generation of valuable co‐products by tuning both anodic and cathodic processes. For example, organic compounds fully oxidized at the anode to CO_2_ can be further reduced at the cathode to form carbon monoxide (CO) or formic acid (HCOOH), making ECAT an attractive strategy for upcycling waste polymers.[^57^, ^58^, ^59^, ^60^] Numerous recent review articles have focused on degradation of other plastics, especially PET via PCAT, ECAT, and photoelectrocatalysis (PECAT).[^13^, ^61^, ^62^, ^63^, ^64^, ^65^, ^66^] However, limited efforts have been made to publish review articles specifically focused on polyolefins (PE and PP) and PS. Therefore, this review aims to evaluate the state‐of‐the‐art in both electro‐ and photo‐catalytic processing of PE, PP, and PS. It begins with a discussion on plastic conversion through solid‐phase PCAT, then transitions to liquid‐phase PCAT, ECAT, and PECAT. The review concludes by outlining strategies to enhance non‐thermal plastic upcycling, discussing catalyst development, and summarizing key findings.

## 2 Solid‐Phase Photocatalysis

Solid‐phase photocatalytic polymer decomposition involves using solid photocatalysts to break down polymers under light irradiation. In this process, a solid photocatalyst (typically a semiconductor metal oxide) is embedded into the plastic material with the aim of promoting the solid‐state degradation of the plastic itself.^[67]^

### 2.1 Historical Developments on Photodecomposition of PE, PP, and PS

In 1974, Allen et al. first reported the photoactivity of anatase TiO_2_ for the decomposition of polyolefins such as PP and PE.^[68]^ They observed that TiO_2_ incorporation into the polymer matrix shifted the excitation wavelength (λ_max_) of carbonyl groups in the polymer to a higher wavelength, making polyolefins more susceptible to decomposition under near‐UV sunlight. Specifically, λ_max_ shifted from 290 to 340 nm for PP and from 270 to–340 nm for PE.

In 1988, Ohtani et al. demonstrated the photodecomposition of PP films loaded with 0.25 wt % TiO_2_ using a 500 W high‐pressure mercury (Hg) lamp with a wavelength of λ<290 nm, in the presence of air at 298 K for 200 h.^[69]^ Transmission electron microscopy (TEM) analysis revealed whitening of the irradiated PP film due to void formation around the TiO_2_ particles, which led to a decrease in mechanical properties and the emission of CO_2_ and CO. The decomposition of PP was attributed to the larger surface area, higher amount of surface ⋅OH, and three orders of magnitude fine particle size of P‐25 TiO_2_ (21 nm) compared to the particle sizes of anatase and rutile TiO_2_ (20 and 120 μm, respectively).

In 1990, Ohtani et al. further explored the solid phase photoinduced oxygenation of low‐density polyethylene (LDPE) and high‐density polyethylene (HDPE).^[70]^ LDPE and HDPE films containing 0.125 to 1.0 wt % TiO_2_ particles were subjected to photoirradiation by a high‐pressure mercury arc lamp. The films experienced physical changes, including whitening, weight loss, and decreased mechanical properties, due to TiO_2_ photocatalytic oxidation. X‐ray diffraction and TEM analyses revealed structural alterations, including the formation of voids in the amorphous regions of PE. Control experiments indicated that the photocatalytic degradation required the co‐presence of TiO_2_ particles, molecular oxygen (O_2_), and light irradiation.

In 2003, Shang et al. investigated the solid‐phase PCAT oxidation of PS using TiO_2_ as the photocatalyst.^[71]^ Both pure PS and PS‐TiO_2_ composite film samples of size 100 cm^2^ were exposed to 8 W UV light with primary wavelength at 253 nm and a light intensity 2.5 mW/cm^2^. The experiments were carried out under ambient air at 298 K. The PS‐TiO_2_ composite exhibited a near doubling of the weight loss compared to pure PS (from 12.0 % to 22.5 % in 150 h). Molecular weight (MW) analysis showed a more pronounced decrease for PS‐TiO_2_ than for pure PS (85 % and 67 %, respectively), indicating enhanced decrease of PE MW through both direct photolytic and PCAT reactions through generation of ROS. Fourier transform infrared spectroscopy (FTIR) analysis detected new C‐O stretching vibration post‐irradiation, indicating chemical changes in the PS composition. Scanning electron microscopy (SEM) analysis revealed the formation of holes and cavities, with PS‐TiO_2_ showing enhanced physical degradation. Gas chromatography (GC) analysis identified volatile organic compounds (VOCs) such as CO_2_, ethene, ethane, and butane released during the degradation. Interestingly, the PS‐TiO_2_ composite produced higher concentrations of CO_2_ (about 230 μM) than the PS system alone (70 μM) in the 18 h reaction.

### 2.2 Major Advances in Plastic Decomposition using TiO_2_

In 2007, Zhao et al. reported ≈42 % weight loss of PE loaded with 1.0 wt % P‐25 TiO_2_ particles under solar radiation and ≈85 % under UV radiation in 300 h.^[72]^ The primary reaction product was CO_2_. The authors proposed that the ROS generated upon photon absorption by TiO_2_ first target the neighboring polymer chains and then diffuse deeper into the polymer matrix, leading to nearly complete polymer decomposition. SEM and TEM analyses revealed cavities in the irradiated PE samples, which increased in size with prolonged solar or UV exposure. However, the decomposition rate of PE did not increase proportionally with the TiO_2_ content. The authors calculated the hole number density, which is directly related to the photocatalytic activity. The hole number densities of 0.10 and 0.02 wt % composite films were directly proportional to the TiO_2_ content, whereas the hole number density of the 1.0 wt % composite film was only 2.5 times that of the 0.1 wt % composite. This was attributed to the agglomeration of TiO_2_ particles at higher loadings, which decreased the interface area between the polymer and TiO_2_, thus decreasing the photodegradation efficiency of the composite film. Several reports further support the photoactivity of TiO_2_ toward PP decomposition owing to either its high surface area or better dispersion of the photocatalyst in the polymer matrix.[^73^, ^74^]

**Figure 5:**
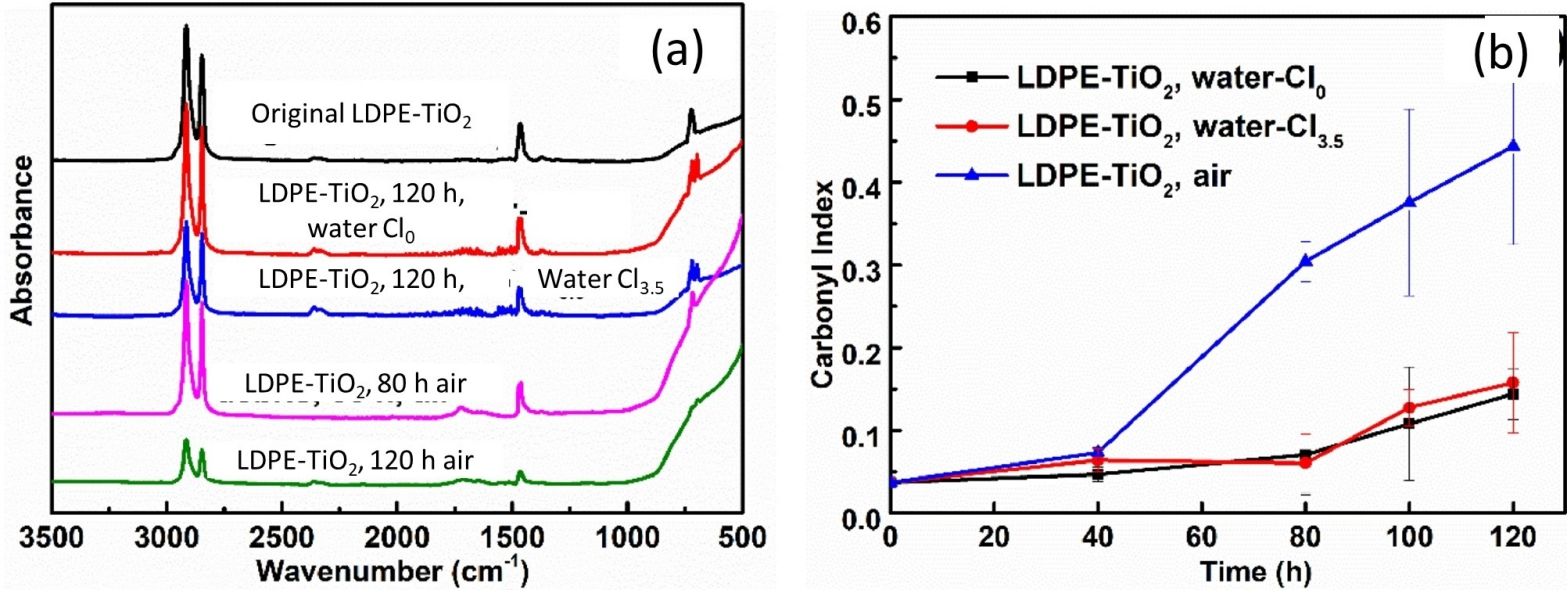
(a) FTIR spectra of the original LDPE‐TiO_2_ film and debris of LDPE‐TiO_2_ film after UV irradiation in different environmental conditions; (b) carbonyl index of the original LDPE‐TiO_2_ film and LDPE‐TiO_2_ in the water without NaCl (water‐Cl_0_), water with 3.5 % of NaCl (water‐Cl_3.5_), and air for 80 h and 120 h irradiation. The figures were adapted from Wang et al.^[75]^ with permission from the publishers.

In 2022, Wang et al. studied the photocatalytic decomposition of LDPE–TiO_2_ film in air and in water with and without NaCl.^[75]^ The experiments were carried out at 24 °C under UV light with primary wavelength at 365 nm and a light intensity of 82 mW/cm^2^. The authors reported ≈80 % mass loss in air as compared to ≈70 % in water with or without NaCl after 120 h of reaction; thus, it appeared that the presence of NaCl did not significantly contribute toward the LDPE decomposition. The increase in the carbonyl content determined via FTIR spectroscopy (Figure 5) in air supported the higher photooxidation of LDPE–TiO_2_ film in air than in water. The authors defined the carbonyl index (CI) as the ratio of the absorbance at the peak of 1716 cm^−1^ (corresponding to carbonyl groups) to the absorbance at the peak of 1465 cm^−1^ (corresponding to methylene groups). From radical trapping experiments, the authors concluded that the photogenerated e^−^ and h^+^ as well O_2_⋅^−^ and ⋅OH were actively participating in the polymer decomposition in the presence of both air and water.

### 2.3 Enhancements and Modifications in Photocatalyst Development for Plastic Photoconversion

Recent advancements have focused on enhancing the performance of wide band‐gap semiconductor PCATs such as TiO_2_ or ZnO under visible light by (1) doping it with metals or non‐metals, (2) coupling it with semiconductors or sensitizers, and (3) exploring other semiconductor metal oxides for improved photocatalytic plastic oxidation.

Verma et al. modified TiO_2_ with reduced graphene oxide (rGO), known to be a good e^−^ acceptor that decreases the rate of recombination of e^−^/h^+^ pairs.^[76]^ The TiO_2_‐rGO photocatalyst was incorporated into the PP matrix and showed enhanced decomposition compared to pristine TiO_2_ after 130 h of solar irradiation. While the authors did not report the extent of decomposition or conversion, they observed the formation of ≈500 nm cavities via SEM analysis and an increase in the carbonyl functionality via FTIR, indirectly confirming the decomposition and functionalization of PP.

**Figure 6:**
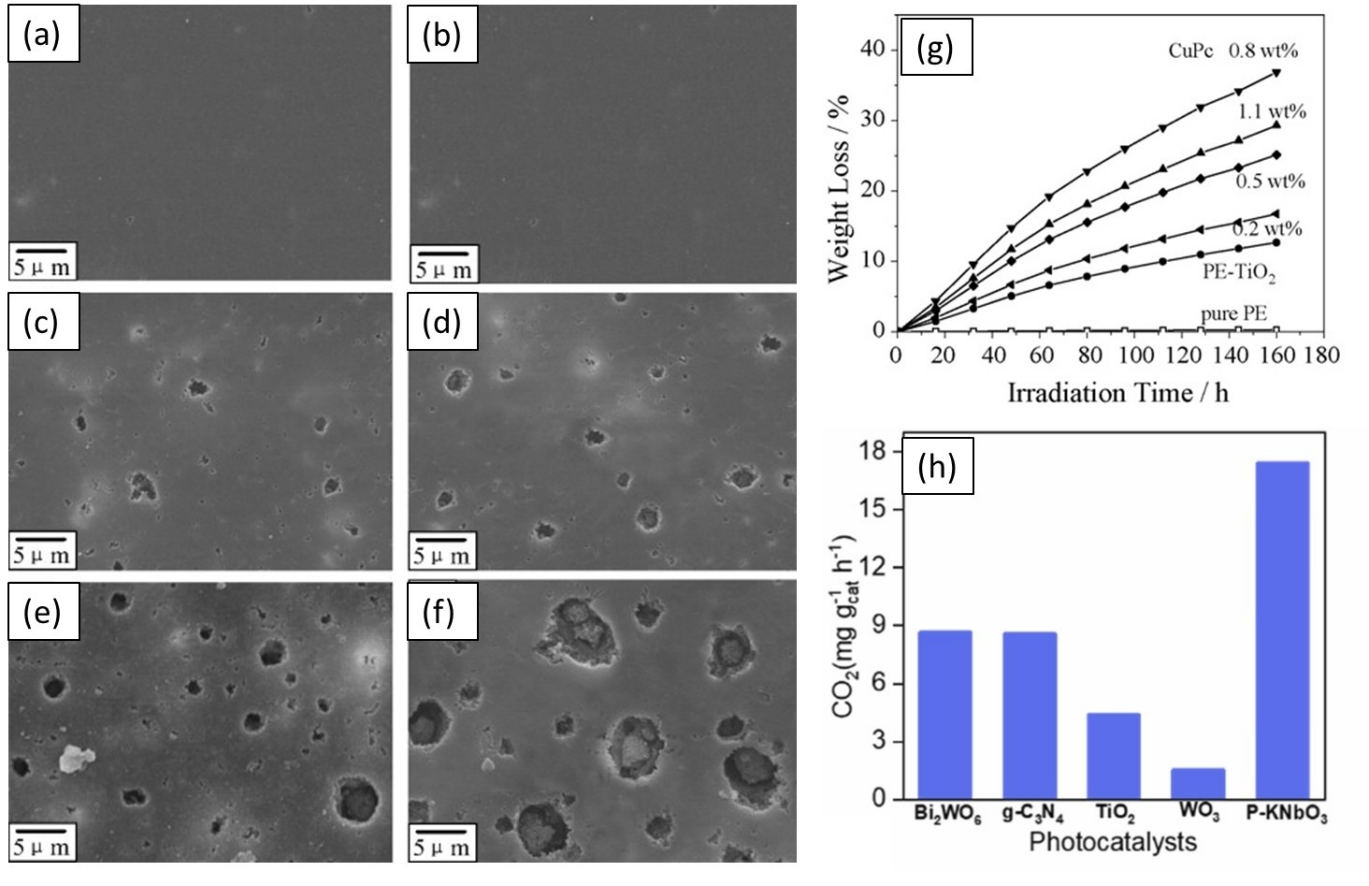
SEM images of PE, PE–TiO_2_, and PE–(TiO_2_/CuPc) films. (a) PE film after solar irradiation for 40 h; (b) PE film after solar irradiation for 80 h; (c) PE–TiO_2_ film after solar irradiation for 40 h; (d) PE–TiO_2_ film after solar irradiation for 80 h; (e) PE–(TiO_2_/CuPc) film after solar irradiation for 40 h; and (f) PE–(TiO_2_/CuPc) film after solar irradiation for 80 h. (g) Weight loss of PE, PE–TiO_2_, and PE–(TiO_2_/CuPc) samples with solar irradiation time. (h) PE photooxidation to CO_2_ over different photo‐catalysts, Bi_2_WO_6_, g‐C_3_N_4_, TiO_2_, WO_3_, P‐KNbO_3_. The figures were adapted from Zhao et al.^[78]^ and Liu et al.^[82]^ with permission from the publishers.

Shang et al. investigated the photoconversion of PS using TiO_2_ with 0.7 wt % of a dye sensitizer, copper phthalocyanine (CuPc).^[77]^ Both PS‐TiO_2_ and PS‐(TiO_2_/CuPc) composite films of size 60 cm^2^ were irradiated using three 8 W fluorescent lamps emitting light between 310 and 750 nm with light intensity 1.75 mW/cm^2^. The experiments were carried out under ambient air at 298 K. UV‐visible absorption spectra indicated that while pure TiO_2_ absorbs UV light (<387 nm), CuPc extends absorption to the visible range (500 to 650 nm), allowing the TiO_2_/CuPc composite to harness a broader light spectrum. Under UV light, TiO_2_ generates e^−^ and h^+^ that can quickly recombine, decreasing PCAT efficiency. The TiO_2_/CuPc composite, however, enables better charge separation. CuPc can be excited by visible light, which promotes charge transfer between TiO_2_ and CuPc, thereby decreasing e^−^/h^+^ recombination and enhancing PCAT activity. The PS‐(TiO_2_/CuPc) composite exhibited a ≈68 % higher weight loss rate than PS‐TiO_2_, 6.9 % and 4.1 % in 250 h respectively. SEM analysis revealed increased cavity and crack formation on PS‐(TiO_2_/CuPc) films post‐irradiation, indicating both surface and bulk photodegradation. GC detected VOCs such as ethene, acetaldehyde, formaldehyde, and ethanol. The PS‐(TiO_2_/CuPc) composite released higher CO_2_ and exhibited lower VOC selectivity compared to PS‐TiO_2_, suggesting enhanced polymer degradation via full oxidation. The PS‐(TiO_2_/CuPc) showed a ≈50 % higher CO_2_ generation rate (36 μM in 22 h) than PS‐TiO_2_. The authors proposed that the degradation involved ROS attacking polymer chains, causing chain scission and oxidation. Effective separation of charge carriers at the CuPc/TiO_2_ interface evaded charge carrier recombination, thus contributing to the more efficient polymer degradation. Similarly, Zhao et al. combined TiO_2_ with 0.8 wt % CuPc and observed an increase in PE weight loss from 10 % to 38 %, along with a rise in CO_2_ generation from 170–27 μM compared to bare TiO_2_.^[78]^ This enhanced PCAT activity was attributed to improved charge separation in TiO_2_/CuPc. SEM images showed minimal cavity density increase in PE–TiO_2_ over time, indicating surface‐focused photodegradation. In contrast, PE–(TiO_2_/CuPc) samples showed much larger and deeper cavities after 40 and 80 h, indicating a higher reaction rate (Figure 6 **a**–**g**). Ali et al. used malachite green as a dye sensitizer coupled with TiO_2_ for LDPE decomposition. They observed a ≈50 % decomposition under visible light within 45 days, which was attributed to enhanced visible light absorption.^[79]^

Lam et al. demonstrated LDPE decomposition using Fe‐doped ZnO.^[80]^ Doping ZnO with Fe decreased its band gap from 3.4 to 3.2 eV. The degradation of LDPE/Fe‐ZnO film was assessed under direct sunlight (271.6 W/m^2^) for 30 days under visible light (57 W/m^2^) using a 105 W fluorescent lamp. Fe‐ZnO nanoparticles were incorporated into the LDPE film, with pure LDPE and LDPE/undoped ZnO as controls. Under sunlight, pure LDPE exhibited 6.1 % weight loss, while LDPE/Fe‐ZnO and showed a ≈7 times higher weight loss (41.3 %) after 120 h. In contrast, LDPE/undoped ZnO only saw a 16.1 % weight loss, 2.6 times lower than when using Fe‐doped ZnO. SEM analysis revealed increased texture changes and deeper cracks in LDPE/Fe‐ZnO after light irradiation, indicating enhanced polymer degradation. Furthermore, FTIR analysis identified new functional groups (unsaturated, peroxide, and carbonyl) in the light‐irradiated LDPE with stronger peaks in LDPE/Fe‐ZnO, confirming its enhanced PCAT activity compared to undoped ZnO. Kemp et al. studied the influence of doping TiO_2_ with transition metals (0.1 to 4.0 wt %) on the photodegradation of PS.^[81]^ While the authors did not provide detailed insights into how these dopants affect the PCAT properties of TiO_2_, they concluded from CI studies via FTIR analysis that the rate of PS photodegradation increased when TiO_2_ was doped with Mo and W ions. In contrast, the rate decreased with the addition of Cr and Mn dopants.

In 2022, Liu et al. investigated the photocatalytic activity of KNbO_3_ and its polarized form (P‐KNbO_3_) for degrading various plastics, including PE, PP, disposable bags, gloves, tubes, and food containers.^[82]^ Polarization decreased the band gap of KNbO_3_ from 3.01 to 2.98 eV and improved the charge carrier lifetime from 8.28 to 8.51 μs, as determined by the time‐resolved photoluminescence. Plastic samples were cut into 1 cm^2^ fragments, combined with photocatalyst KNbO_3_ or P‐KNbO_3_, and tested in an infrared reaction cell with air and 86 % humidity, using a 150 W xenon lamp (25 mW/cm^2^). P‐KNbO_3_ showed 210 % and 140 % improvements in photocatalytic efficiency for PE and PP decomposition, respectively, compared to KNbO_3_. P‐KNbO_3_ also performed well with real‐world waste plastics, including bags, gloves, tubes, and boxes made of PE or PP. Compared to traditional photocatalysts such as Bi_2_WO_6_, g‐C_3_N_4_, TiO_2_, and WO_3_, P‐KNbO_3_ exhibited 2 to 9 times higher activity for PE conversion (Figure 6h). The authors proposed that generated ⋅OH and O_2_⋅^−^ radicals under air and H_2_O atmosphere, detected via electron spin resonance (ESR), increased the CO_2_ production by ≈11 times. These radicals and photogenerated h^+^ attack the C‐H bonds of PE and PP, and form carbon‐centered radicals that oxidize to carbonyl and carboxyl compounds, which are eventually converted to CO_2_ and H_2_O.

Solid‐phase PCAT usually incorporates TiO_2_, ZnO, or KNbO_3_ into the polymer matrix to decompose plastics under light irradiation. While historical advancements have improved the efficacy of these methods by developing new materials, additional work is required to (1) assess the long‐term stability and reusability of these PCATs as well as (2) perform a techno‐economic analysis to benchmark PCAT processes against the commercial technologies for plastic decomposition.

## 3 Liquid‐Phase Photocatalysis for Plastics: PE, PP, and PS

In liquid‐phase PCAT, photoactive semiconductor nanoparticles are either (1) dispersed in an aqueous medium to break down plastics under light irradiation or (2) placed onto the surface of a plastic film to promote the degradation of contaminants in an aqueous medium. Once the nanoparticles are irradiated, excitation of the semiconductor leads to the generation of ROS (O_2_⋅^−^ and ⋅OH), which attack the targeted substrates and decompose them.

### 3.1 Initial Observations and Advances in Plastic Conversion via Liquid‐Phase Photocatalysis

In 2019, Tofa et al. investigated decomposition of LDPE microplastics using ZnO nanorods as photocatalysts under visible light.^[83]^ The experiment ran for 175 h under a 50 W halogen lamp (60 to 70 klux), with 1 cm^2^ LDPE film in a petri dish containing water and a photocatalyst. Increased stiffness, cracks, and chemical changes in the plastic films were confirmed by dynamic mechanical analyzer and time–dependent FTIR analyses. The authors monitored the effect of photocatalytic oxidation by determining CI and vinyl index (VI). Higher CI and VI values suggest higher extent of oxidation. The CI is calculated as the ratio of areas under the absorbance peaks at 1712 and 1372 cm^−1^, and the VI is calculated as the ratio of the area under the absorbance of the vinyl group at 909 cm^−1^ to the area under the same reference peak. A 112 % increase in CI and 156 % increase in VI were observed from photooxidation of LDPE‐ZnO compared to pristine LDPE. Higher PCAT efficiency was associated with the increased surface area of longer nanorods. The authors suggested that O_2_⋅^−^ and ⋅OH generated from the PCAT under light irradiation initiate the decomposition at weak spots in the polymeric chains such as chromophoric groups (parts of the polymeric chain that absorb light and become reactive), leading to the formation of low MW PE alkyl radicals. This is followed by chain breaking, crosslinking, and oxidation. The formation of HOO⋅ and ROOH further facilitates oxidative decomposition, eventually producing carbonyl (C‐O) and vinyl (RCH‐CH) group‐containing species, chain cleavage, and VOCs like ethane and formaldehyde, with further oxidation potentially leading to complete mineralization to CO_2_ and water.

**Figure 7:**
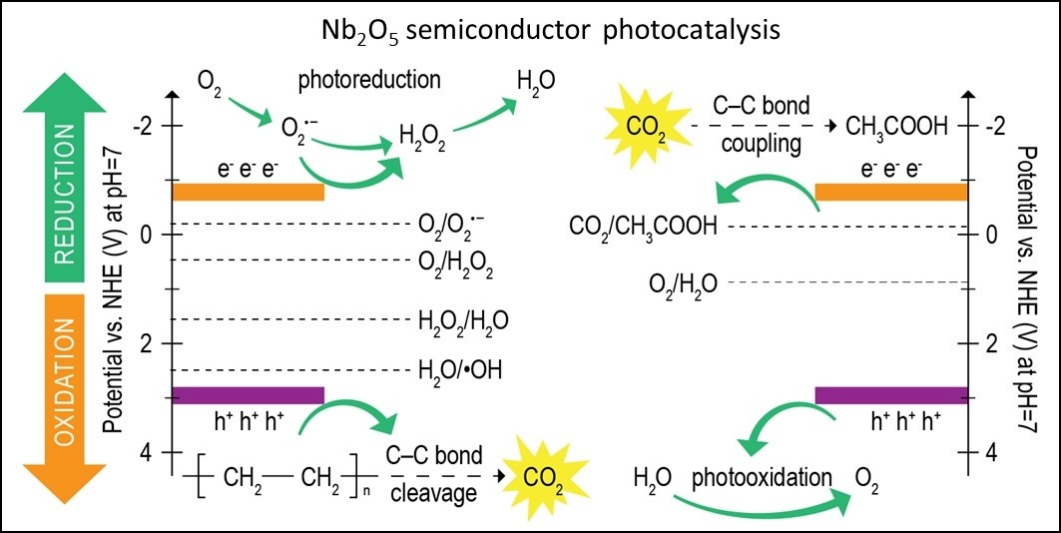
Schematic representations of the band edge positions of Nb_2_O_5_ along with the potentials of CO_2_, H_2_O, H_2_O_2_, and O_2_ redox couples at pH 7 and the proposed two‐step C‐C bond cleavage and coupling mechanism from pure PE into CH_3_COOH under simulated natural environment conditions. The figure is adapted from Jiao et al.^[84]^

In 2020, Jiao et al. fabricated Nb_2_O_5_ monolayer nanosheets to completely degrade PE and PP into CO_2_ and acetic acid in 40–60 h.^[84]^ The experiments used the reference spectrum for standard testing of solar energy conversion of AM 1.5 G at room temperature and atmospheric pressure. Nb_2_O_5_ photocatalyst is a wide band gap semiconductor with VB maximum at 2.62 V vs. a normal hydrogen electrode (NHE) at pH 7, which is strong enough to generate the powerful ⋅OH radicals from water (e. g., H_2_O→H^+^+⋅OH at 2.2 V). On the other hand, this photocatalyst with a CB minimum at −0.9 V can reduce O_2_ to O_2_⋅^−^ radicals and H_2_O_2_ and reduce CO_2_ to acetic acid (e. g., CO_2_+4H^+^+2 e^−^→ CH_3_COOH at −0.6 V vs. NHE). A schematic representation is shown in Figure 7. The authors monitored photodegradation of PE via in situ electron paramagnetic resonance, isotope labeling, and UV‐visible absorption. They concluded that the photoexcited h^+^ in the VB of Nb_2_O_5_ produced ⋅OH, which was responsible for the C‐C cleavage of PE into CO_2_. Simultaneously, the photogenerated e^−^ in the CB mainly participated in the reduction of O_2_ into O_2_^.−^ and H_2_O_2_. From in situ FTIR studies, the authors proposed that a portion of the photogenerated e^−^ participated in the reduction of CO_2_ to acetic acid with a 0.2 % yield. The low yield of acetic acid was attributed to the poor adsorption of the reaction intermediates (e. g., HOOC‐CO⋅) and desorption of acetic acid according to the density functional theory calculations.

In 2020, Lorente‐García et al. demonstrated the photoactivity of nitrogen‐doped TiO_2_ for decomposing LDPE and HDPE microplastics under visible light.^[85]^ Doping TiO_2_ with N slightly decreased its band gap from 3.2 to 3.1 eV. The study highlighted that both the size and shape of the microplastics impact the effectiveness of the degradation process. The mass loss from 5 mm LDPE films was 0.97 %, compared to a 1.38 % mass loss from 3 mm LDPE films. Smaller microplastics were more easily degraded than larger ones due to their increased surface area, which enhances the PCAT reaction. The shape of the microplastics also influenced the degradation efficiency, with irregularly shaped particles exhibiting different degradation rates compared to spherical particles. HDPE microplastics were spherical while LDPE microplastics were in the form of films. The degradation rate constant for spherical HDPE (3.8 mm) was ≈14.2×10^−4^ h^−1^, whereas for LDPE film (3.0 mm), the rate constant was ≈4.02×10^−4^ h^−1^.

**Figure 8:**
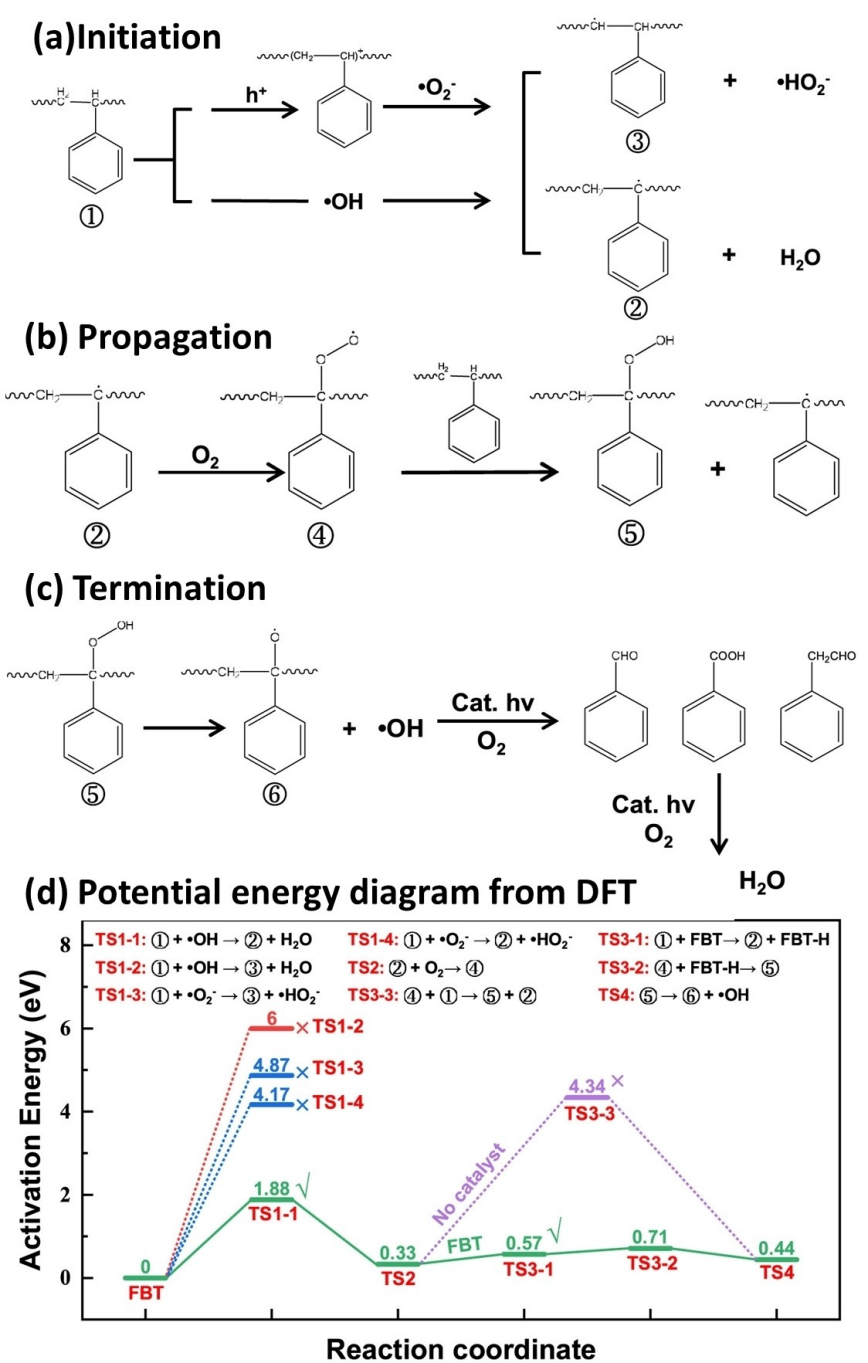
The pathway of photocatalytic polystyrene (PS) degradation; (a) chain initiation, (b) propagation, (c) termination, and (d) Potential energy diagram from density functional theory calculations of PS microplastics degradation. Note that TS is short for transition state. The figures are adapted from He et al.^[86]^ with permission from the publisher.

In 2023, He et al. enhanced the visible light absorption of TiO_2_ by alloying it with FeB, creating a photocatalyst composite (FeB/TiO_2_) for converting PS microparticles into H_2_ and valuable organic compounds such as toluene, styrene, benzaldehyde, phenylacetaldehyde, and benzoic acid.^[86]^ The band gap of TiO_2_ decreased from 3.21 to 2.63 eV in FeB/TiO_2_. The TiO_2_ core‐shell structure was synthesized via alcoholysis, while FeB‐modified TiO_2_ was synthesized by reducing iron sulfate with NaBH_4_. X‐ray diffraction confirmed the TiO_2_ anatase phase and the dispersion of FeB, while SEM and TEM images revealed the core‐shell structure. UV‐visible diffuse reflectance spectroscopy demonstrated enhanced light absorption, and steady‐state fluorescence showed decreased charge carrier recombination. The authors proposed a possible degradation pathway for PS‐microplastics as depicted in Figure 8a–c. During the chain initiation process shown in Figure 8a, O_2_^.**−**^ and ⋅OH abstract hydrogen from α or β carbons connected to the benzene ring of PS molecules, forming intermediates 2 and 3. Density functional theory calculations show that forming intermediate 2 requires lower activation energies (1.88 eV for ⋅OH and 4.17 eV for O_2_^.−^) compared to intermediate 3 (6 eV for ⋅OH and 4.7 eV for O_2_^.−^), as seen in Figure 8d. This suggests the α‐carbon connection is more susceptible to activation, especially by ⋅OH. In the chain propagation step (Figure 8b), intermediate 2 integrates oxygen to form intermediate 4 (transition state [TS] 2, 0.33 eV) but has a high barrier for hydrogen abstraction from another PS molecule (TS3‐3, 4.34 eV). Addition of FeB/TiO_2_ splits this into two low‐energy steps: (1) FeB/TiO_2_ abstracts hydrogen to form intermediate 2 (TS3‐1, 0.57 eV) then (2) combines with intermediate 4 to form intermediate 5 (TS3‐2, 0.71 eV), facilitating rapid chain propagation. Finally, a hydroxyl group is abstracted from intermediate 5 to produce intermediate 6 (TS4, 0.44 eV), leading to benzaldehyde, phenylacetaldehyde, and benzoic acid via photocatalytic oxidation (Figure 8c). The FeB/TiO_2_ photocatalyst facilitated these reactions by lowering activation energies and enhancing e^−^ transfer, leading to the complete oxidation of some intermediates to CO_2_ and enabling H_2_ evolution, thus integrating energy recycling with pollutant degradation.

**Figure 9:**
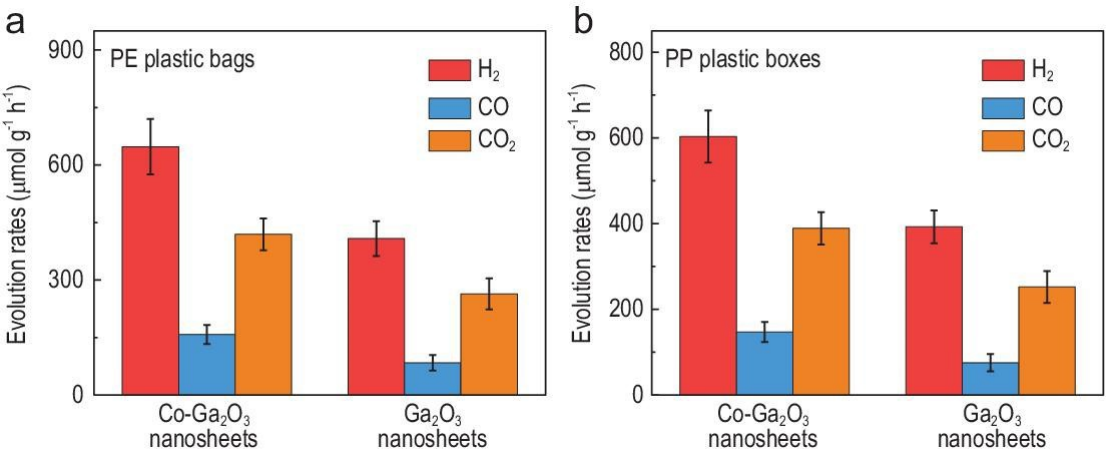
Efficient photoconversion of various plastics into syngas over Co–Ga_2_O_3_ nanosheets under mild conditions. Photoconversion of (a) commercial PE plastic bags, (b) commercial PP plastic boxes. The figures are adapted from Xu et al.^[87]^ with permission from the publisher.

In 2022, Xu et al. demonstrated the photocatalytic conversion of commercial plastic products (e. g., PE bags, PP boxes, and PET bottles shredded into powders with a size of <5 mm) into renewable syngas using Co‐Ga_2_O_3_ nanosheets.^[87]^ These plastics were dispersed in pure water at ambient conditions and exposed to simulated sunlight (AM 1.5 G, 100 mW/cm^2^). Photogenerated charge carriers converted H_2_O to H_2_ and O_2_, while ⋅OH photodegraded plastics into CO_2_, which was further photoreduced into CO via ✶COOH intermediates. Weight losses for PE, PP, and PET were 81 %, 78 %, and 72 %, respectively, after 48 h of irradiation with Co‐Ga_2_O_3_, compared to 30 %, 29 %, and 26 %, respectively, for bare Ga_2_O_3_. The conversion of PE and PP produced H_2_, CO, and CO_2_ at higher rates with Co‐Ga_2_O_3_ than with bare Ga_2_O_3_, as illustrated in Figure 9. Using commercial PE plastic bags as an example, the Co‐Ga_2_O_3_ nanosheets generated H_2_, CO, and CO_2_ at 647.8, 158.3, and 419.3 μmol/(g ⋅ h), respectively, ≈1.6, 1.9, and 1.6 times higher than those of the Ga_2_O_3_ nanosheets. Increased photoactivity was attributed to a decrease in band gap (from 4.65 to 3.90 eV), enhanced photocarrier transfer, and suppression of photogenerated e^−^ and h^+^ recombination with Co introduction. Co‐Ga_2_O_3_ also showed stronger CO_2_ adsorption behavior, facilitating further CO_2_ reduction to CO.

### 3.2 Enhanced Photocatalytic Systems for Plastic Upcycling

**Figure 10:**
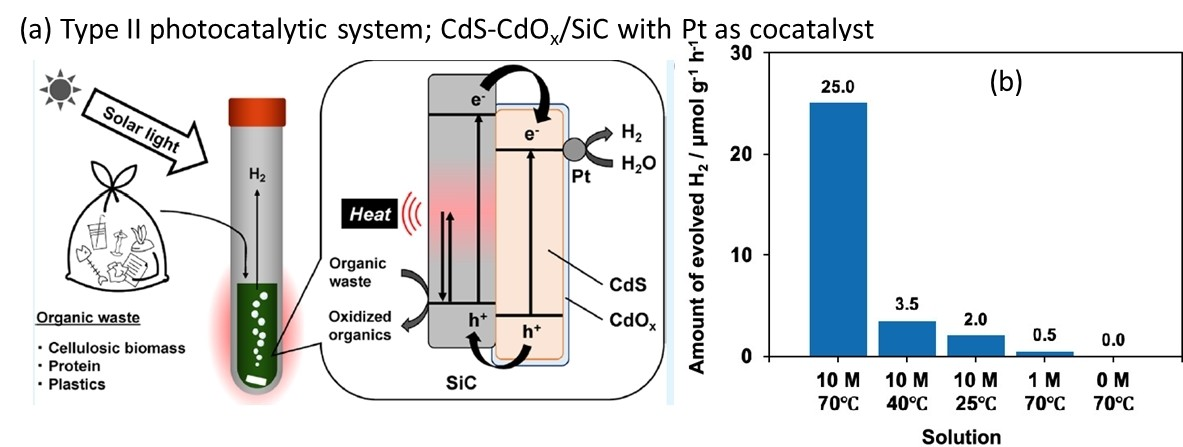
Schematic illustration of the photoreforming of organic waste into molecular hydrogen (H_2_) using Pt‐deposited CdO_x_/CdS/SiC composite photocatalyst. (b) Effects of temperature and basicity of the NaOH reaction solution on rate of H_2_ production from PE photoreforming. The figures were adapted from Nagakawa et al.^[88]^ with permission from the publisher.

In 2021, Nagakawa et al. created a CdS‐CdO_x_/SiC, type II e^−^ transfer photocatalytic system with 0.5 wt % Pt as a cocatalyst for photoreforming of organic waste, including LDPE.^[88]^ In a typical type II photocatalytic system, the e^−^ from the CB of the first semiconductor migrate to the CB of the second semiconductor.^[89]^ Correspondingly, the photogenerated h^+^ in the VB of the second semiconductor migrate to the VB of the first semiconductor.^[89]^ This charge separation enhances photocatalytic reactions. In the CdS‐CdO_x_/SiC system, SiC serves as the first semiconductor and CdS‐CdO_x_ as the second, based on their respective VB and CB positions. A schematic representation of this system is shown in Figure 10a. The collected e^−^ in the VB of CdS‐CdO_x_ are further transported to the Pt co‐catalyst, facilitating hydrogen evolution reaction. Meanwhile, h^+^ in SiC directly participate in the oxidation of organic compounds and plastics. This combination of semiconductors in a type II system delays e^−^/h^+^ recombination, increasing photocatalytic efficiency. This system was used for the photoreforming of LDPE.^[88]^ The experiments were conducted in a 32 mL tube with 100 mg of substrate and 50 mg of photocatalyst suspended in 10 M NaOH using a 300 W Xe lamp. The H_2_ production from LDPE increased with increase in temperature and NaOH concentration, reaching ≈25 μmol/(g_cat_ ⋅ h) at 70 °C and 10 M NaOH as shown in Figure 10b. The decomposition of PE gloves under simulated sunlight simultaneously produced H_2_ at 0.72 μmol/(g_cat_ ⋅ h). This study shows the capability of type II photocatalytic systems for plastic decomposition; however, there is a need for more eco‐friendly photocatalytic systems to avoid the use of heavy metals like Cd for sustainable plastic reforming applications.

**Figure 11:**
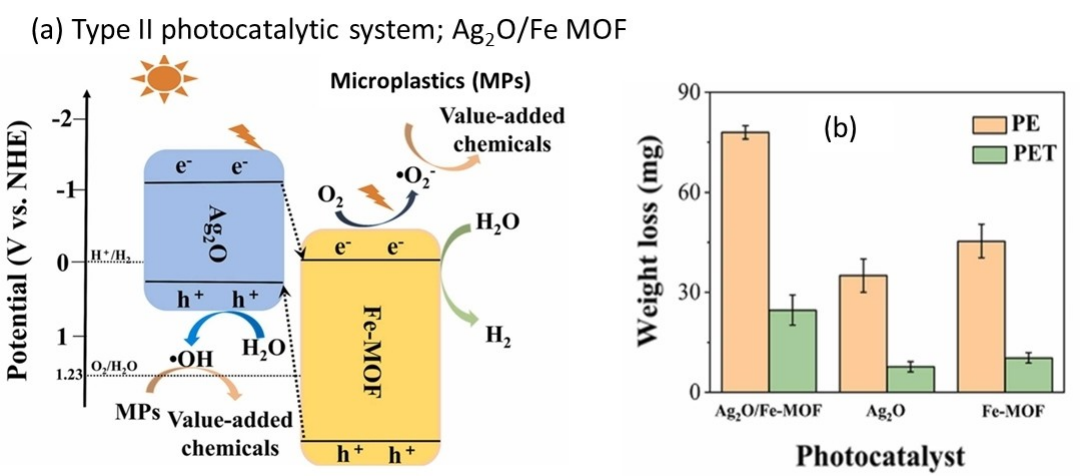
(a) Schematic illustration of Ag_2_O/Fe‐MOF heterojunction photocatalyst and the application in upcycling various PET and PE into value‐added chemicals and sustainable fuel H_2_. (b) The weight loss from PE and PET conversion from photocatalysts Ag_2_O, Fe‐MOF, and Ag_2_O/Fe‐MOF, respectively. The figures were adapted from Qin et al.^[90]^ with permission from the publisher.

In 2022, Qin et al. also demonstrated the decomposition of plastics using a type II visible light active photocatalytic system (Figure 11a).^[90]^ They developed an Ag_2_O/Fe metal‐organic framework (MOF) by incorporating Ag_2_O particles into Fe‐based MOF pores, enhancing heterojunction formation and charge separation, as confirmed by photoluminescence and ESR studies. Pristine Ag_2_O and Fe‐MOF exhibited low photocatalytic activity due to their wide band gaps (Figure 11b). In contrast, Ag_2_O/Fe‐MOF showed improved photocatalytic performance for PE and PET decomposition, likely due to better visible light absorption. H_2_ production with Ag_2_O/Fe‐MOF increased approximately 17‐fold compared to Ag_2_O and 1.7‐fold compared to Fe‐MOF. Acetic acid was a major carbon product from polyethylene glycol (PEG), but no carbon products from PET or PE decomposition were reported. While Ag_2_O/Fe‐MOF showed stable PET decomposition over five cycles, the detailed mechanism for PE decomposition remains unclear.

**Figure 12:**
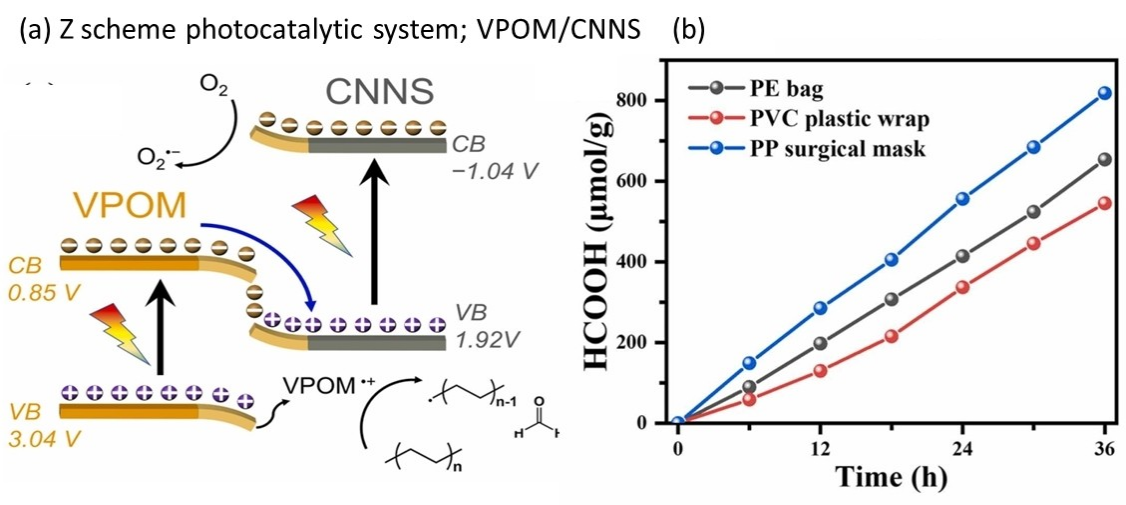
Schematic of photocatalytic plastic upcycling process for VPOM/CNNS Z‐scheme heterojunction. (b) HCOOH production from photocatalytic upcycling of real‐world plastic waste over VPOM/CNNS‐15. The figures were adapted from Xing et al.^[91]^ with permission from the publisher.

Xing et al. developed a Z‐scheme photocatalytic system with vanadium substituted phosphomolybdic acid/g‐C_3_N_4_ nanosheets (VPOM/CNNS) for upcycling PE and PP to formic acid.^[91]^ They prepared VPOM/CNNS hybrids with varying VPOM mass (10 to 25 mg) while keeping CNNS constant at 100 mg. Among these, VPOM/CNNS‐15 exhibited a photo response up to five times higher than pristine CNNS and other VPOM/CNNS‐X hybrids. Additionally, the average charge carrier lifetime in VPOM/CNNS‐15 was longer (17.84 ns) compared to pristine CNNS (12.84 ns), indicating improved charge separation and enhanced photocatalytic activity. Under visible light (λ>420 nm), VPOM/CNNS achieved a formic acid production rate of 24.7 μmol/(g_cat_ ⋅ h), approximately 262 times higher than that of CNNS alone. No formic acid was produced from pristine VPOM. The photocatalyst maintained stability for 100 h during PE upcycling and successfully converted various real plastics, including PE bags, PVC wraps, and PP surgical masks, into formic acid (Figure 12b). The Z‐scheme heterojunction in VPOM/CNNS was confirmed through transient absorption spectroscopy, in situ X‐ray photoelectron spectroscopy, and ESR measurements, providing insight into the charge transport process. UV photoelectron spectroscopy and diffuse reflectance spectroscopy helped determine the band gap and energy levels of VPOM and CNNS. Using data from various characterizations and density functional theory, the authors proposed a reaction mechanism for plastic photoconversion shown in Figure 12a.^[91]^ Under light irradiation, the photogenerated e^−^ in the CB of VPOM combine with the h^+^ in the VB of CNNS, a typical phenomenon in a Z‐scheme heterojunction process enhancing charge separation. This leads to the accumulation of e^−^ in the CB of CNNS, reducing O_2_ to O_2_⋅^−^ radicals. Meanwhile, the h^+^ in the VB of VPOM perform oxidative C‐C cleavage converting PE into formaldehyde, which is further oxidized to formic acid by O_2_⋅^−^ radicals. Despite a low efficiency (0.44 %) for PE upcycling, this study uniquely demonstrates the potential for Z‐scheme heterojunction photocatalysts in plastic upcycling.

### 3.3 Coupled Plastic Chemical Depolymerization with Photocatalytic Upcycling

**Figure 13:**
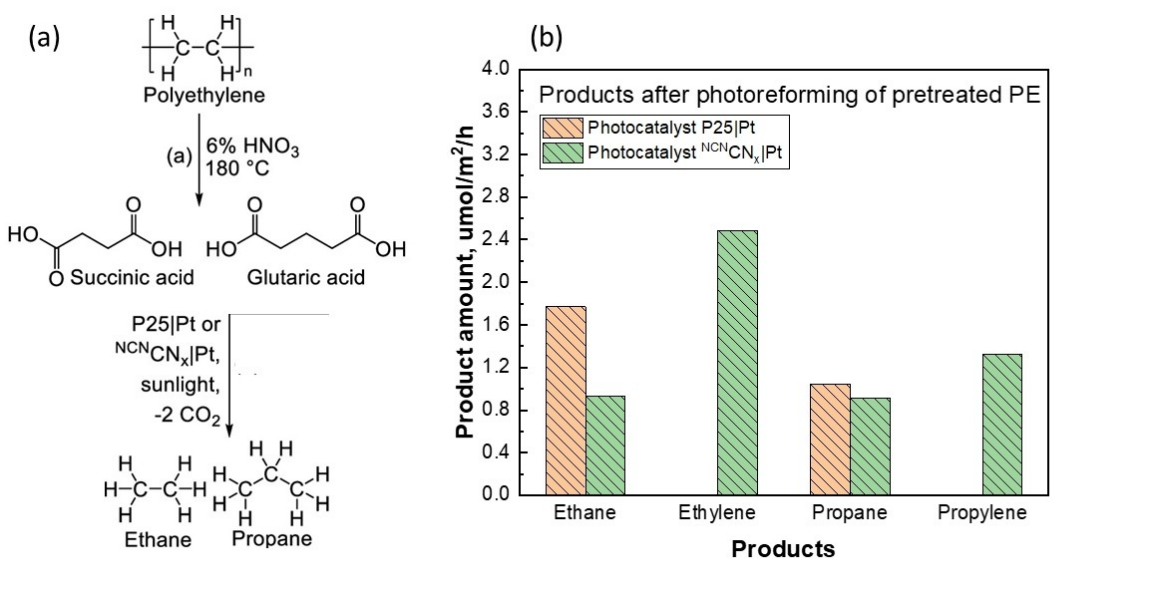
(a) Schematic for oxidative PE conversion to dicarboxylic acids, which can subsequently be converted into gaseous hydrocarbon products via photocatalysis to give alkanes. (b) Product yields from photocatalytic experiments with TiO_2_|Pt and ^NCN^CN_x_|Pt deposited on glass sheets. Conditions: AM 1.5 G, 100 mW/cm^2^, backside irradiation, 25 °C, and 50 mL of PE decomposition solution. The figures were adapted from Pichler et al.^[55]^ with permission from the publisher.

In 2021, Pichler et al. developed a two‐step process to convert PE into value‐added products. Initially, PE was oxidized with 6.0 wt % HNO_3_ at 180 °C for 4 h.^[55]^ This step achieved complete PE conversion, yielding succinic acid (44 %) and glutaric acid (22 %) as major products. In the second step, the PE decomposed solution containing the acid products was converted to either (1) alkanes via PCAT **(**Figure 13a) or (2) alkenes via ECAT. For the photocatalytic route, two photocatalysts were tested: (1) UV active TiO_2_ P25 (known simply as P25) and (2) visible light active cyanamide modified carbon nitride (^NCN^CN_x_). These catalysts were loaded with 1.0 wt % Pt. Prior to testing these photocatalysts for PE decomposed solution, they were tested for photocatalytic conversion of succinic acid (10 ppm) as a model compound in 0.1 M HNO_3_. The experiments were carried out in a sealed glass batch reactor under simulated solar light in a nitrogen atmosphere at 25 °C for 24 h. Ethane was the primary hydrocarbon product. The authors proposed that photogenerated h^+^ were responsible for the oxidative decarboxylation of succinic acid to propanoic acid, which was further converted to ethane. Other products included H_2_ and CO_2_, with ethylene observed as a secondary product using the ^NCN^CN_x_/Pt catalyst. When glutaric acid served as the next model substrate, propane and propylene were the major products, formed via butyric acid as an intermediate. The authors then performed photocatalytic experiments with actual PE decomposed solution. To counteract reduced light absorption due to the yellow color of the pure PE decomposed solution, the solution was diluted to 10 : 1 with water before PCAT. Ethane, ethylene, propane, and propylene were the major hydrocarbon products, with an overall yield of 1.0 % and approximately 6.1 % for CO_2_. Additional experiments using a flow setup with a photocatalyst panel deposited on a glass sheet demonstrated continuous product generation over a 72 h reaction period. The rates of product formation from photocatalysts P25/Pt and ^NCN^CN_x_/Pt are shown in Figure 13b. This flow system addressed the challenges of decomposing colored PE solutions in a batch system, showing direct conversion viability via PCAT. Further studies on this system under electrochemical conditions provided additional insights, as described in the ECAT section.

**Figure 14:**
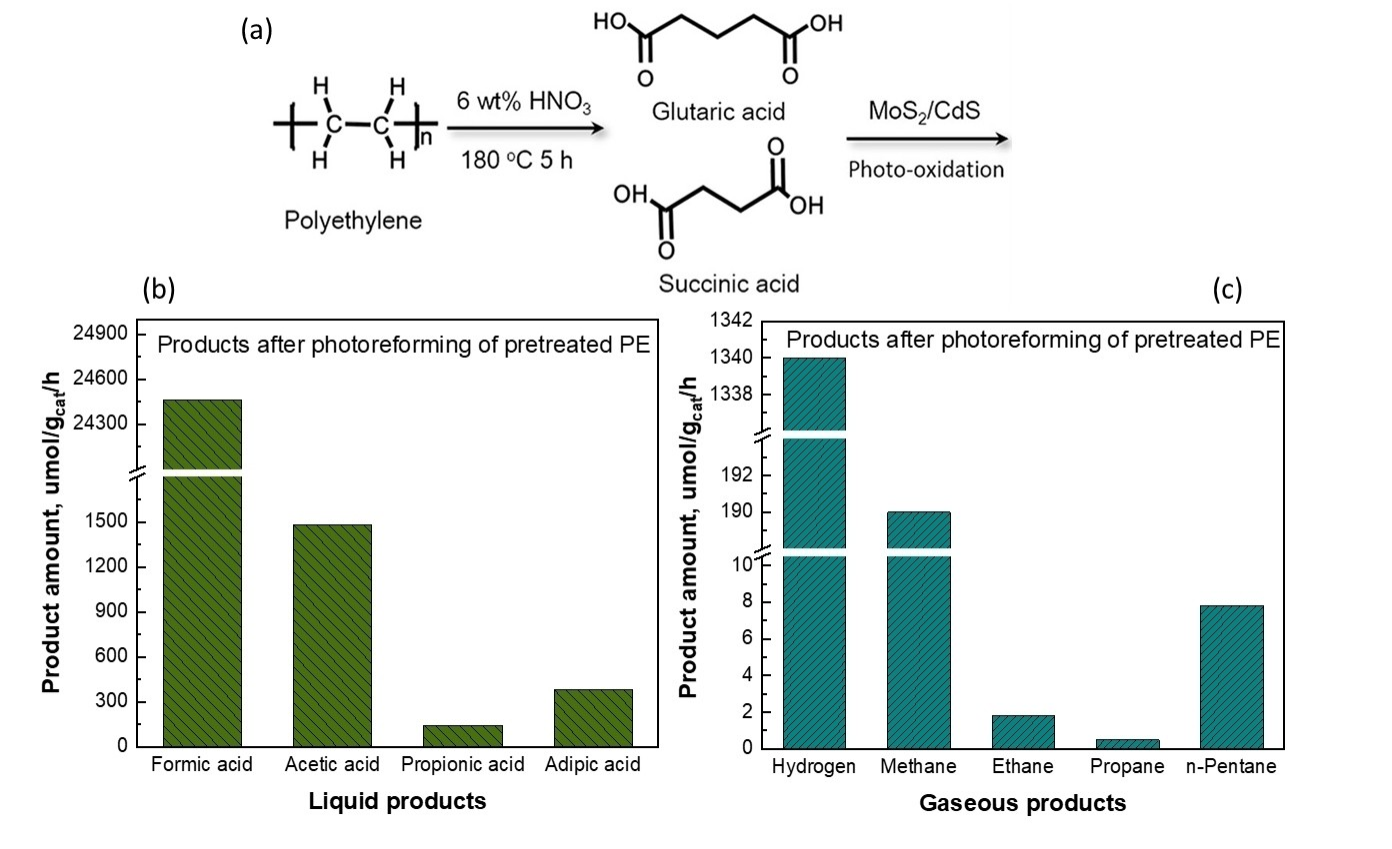
(a) Schematic illustration of PE oxidation into carboxylic acid and the subsequent photoreforming process over MoS_2_/CdS. Products from photoreforming of pretreated PE under simulated solar light over photocatalyst MoS_2_/CdS: (b) liquid products, (c) gaseous products. The figures were adapted from Du et al.^[92]^ with permission from the publisher.

Similar to Pichler et al., Du et al. in 2022 also demonstrated a two‐step process to upcycle PE.^[92]^ The first step is identical, where PE is converted into carboxylic acids using nitric acid, yielding succinic acid and glutaric acid. These pretreated plastics were then subjected to photoreforming. A schematic of this process is shown in Figure 14. The group used MoS_2_‐tipped CdS nanorod photocatalysts in an aqueous medium to reform pretreated plastics. Electrons are collected at the MoS_2_ tips for H_2_ evolution, while the entire sidewalls of the CdS nanorods, rich in h^+^, facilitate the oxidation of plastics. The photoreforming process produced formic acid as the main liquid product (Figure 14b), and substantial amounts of H_2_, which mainly originated from water splitting with a stable rate of 1.13±0.06 mmol/(g_cat_ ⋅ h), and maintained this activity over 200 h (Figure 14c). MoS_2_/CdS also generated CH_4_ at 196.2±1.76 μmol/(g_cat_ ⋅ h) and CO_2_ as a byproduct. CH_4_ predominantly resulted from carboxylic acid decarboxylation rather than CO_2_ reduction. Other alkanes, including ethane, propane, and n‐pentane, were produced via similar decarboxylation reactions. In situ ESR spectroscopy confirmed that MoS_2_/CdS effectively decarboxylates both monocarboxylic and dicarboxylic acids. This strategy of using MoS_2_/CdS photocatalyst holds potential for generating valuable chemicals while addressing plastic waste management. However, it does not show the long‐term durability of the catalyst and potential for deactivation over extended use. Also, yield and selectivity of the products are not reported. The study lacks a thorough comparison of MoS_2_/CdS with other photocatalysts, including an evaluation of why it outperforms or underperforms compared to alternative materials.

Zhang et al. in 2023 reported a similar two‐step process to convert PE using TiO_2_‐supported atomically dispersed Pd species.^[93]^ The authors first conducted photocatalytic experiments using succinic, the main product of hydrolyzed PE solution, as the model compound. The CO_2_ yield from Pd‐TiO_2_ increased by 345 % (0.49 mmol) compared to TiO_2_ (0.11 mmol). The increase in photocatalytic conversion was attributed to the accumulation of more photo‐generated charges on the Pd‐TiO_2_ surface compared to bare TiO_2_ as determined from transient surface photovoltage spectroscopy. Suppression of the e^−^/h^+^ recombination was evident from transient photoluminescence studies. Pd‐TiO_2_ could convert PE hydrolyzed solution into different products such as propionic acid at 265.5 μmol/(g_cat_ ⋅ h), ethane at 32.7 μmol/(g_cat_ ⋅ h), and ethylene at 23.3 μmol/(g_cat_ ⋅ h). The authors proposed that after generating e^−^/h^+^ pair during the photoexcitation, Pd sites trapped e^−^, forming Pd^δ+^ species, while TiO_2_ h^+^ and ⋅OH drive oxidative decarboxylation of PE solution (containing mainly succinic acid) to propionic acid radicals, which are either hydrogenated to propionic acid or stabilized for decarboxylation into ethane or ethylene via Pd‐mediated decarboxylation and homolytic Pd‐C bond cleavage.

## 4 Electrocatalysis of Plastics

The ECAT route has rarely been explored for the upcycling of waste plastic, and the majority of the reports focus on PET upcycling.[^61^, ^62^, ^63^] Remarkably, we found only one publication each addressing the electrocatalytic upcycling of PE and PS, underscoring the need for further research to tackle the decomposition of the most abundant and recalcitrant waste polymers, namely PE, PP, and PS.

**Figure 15:**
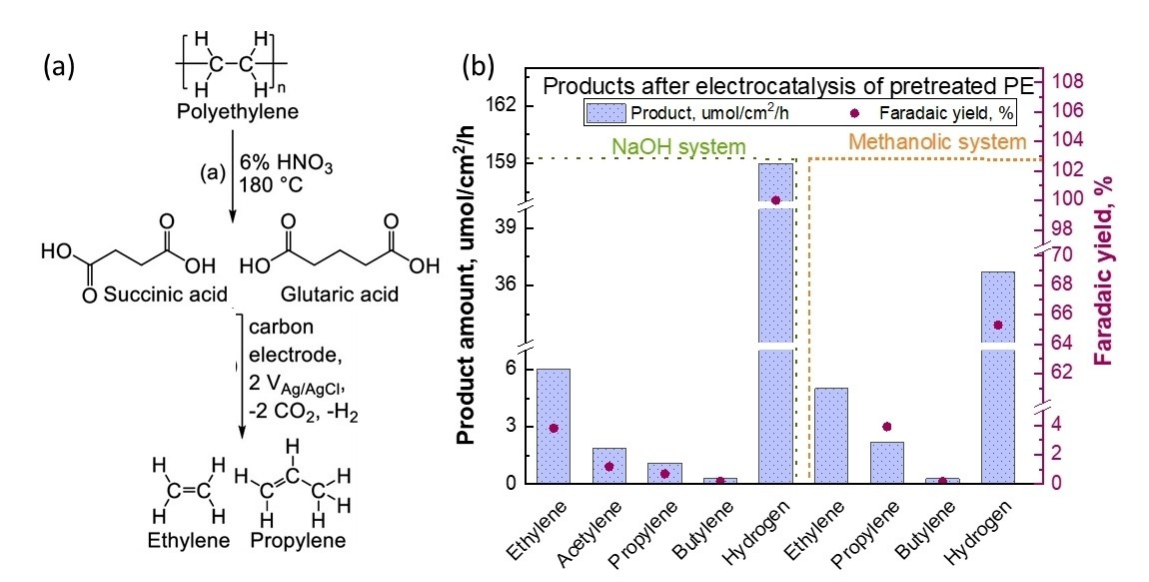
(a) Schematic of oxidative PE conversion to dicarboxylic acids, which can subsequently be converted into hydrocarbon products via electrolysis to produce alkenes. (b) Product yields from chronoamperometric tests. Reaction conditions: carbon paper as working electrode and Pt foil as counter each with 2 cm^2^ electrode area, applied voltage 5 V, reaction time until 130±25 Coulomb passed. Two different solvents tested. First is aqueous NaOH system which had 8 mL PE decomposition solution and 16 mL H_2_O set to pH 10 with 10 M NaOH. Second is methanol system where 8 mL of PE decomposition solution was diluted with 16 mL methanol. The figures were adapted from Pichler et al.^[55]^ with permission from the publisher.

Pichler et al. demonstrated a two‐step process to upcycle PE. First, the PE was depolymerized in presence of HNO_3_ at 180 °C to form PE decomposed solution containing mainly succinic acid and glutaric acid.^[55]^ The carboxylic acids were then converted to alkenes via the electrocatalytic route; a schematic of this process is shown in Figure 15a. The electrochemical experiments were conducted with an Ivium CompactStat potentiostat. A two‐electrode setup was used, with carbon paper as the working electrode and Pt foil as the counter electrode. The electrochemical cell included additional headspace volume (130 mL glass bubble) to accommodate larger amounts of gaseous products. About 24 mL of PE solution was purged with N_2_ for 20 min before experimentation. A voltage of 5 V was applied, achieving current densities of approximately 20–30 mA/cm^2^. Reaction products were monitored by manually sampling and analyzing the headspace (50 μL) via GC and HPLC. The authors tested PE solution in two different electrolyte systems: (1) a mixture of a methanol water solvent system (2 : 1 MeOH: H_2_O solution at pH 4) and (2) an aqueous NaOH system with pH 10. In the NaOH system, ethylene, acetylene, propylene, and butylene with Faradaic yields of 4.0 %, 1.4 %, 0.7 %, and 0.2 % with product formation rate of 6.0, 1.9, 1.1, and 0.3 μmol/(cm^2^ ⋅ h), respectively, were detected, (Figure 15b). In the methanolic system, ethylene, propylene, and butylene with Faradaic yields of 9.0 %, 3.9 %, and 0.2 % with product formation rates of 5.0, 2.2, and 0.3 μmol/(cm^2^ ⋅ h), respectively, were detected. H_2_ was produced at the cathode in both cases. While the work does not show direct electrocatalytic plastic upcycling, it demonstrates a hybrid, two‐step approach to electrochemically converting PE into value‐added olefins and H_2_.

**Figure 16:**
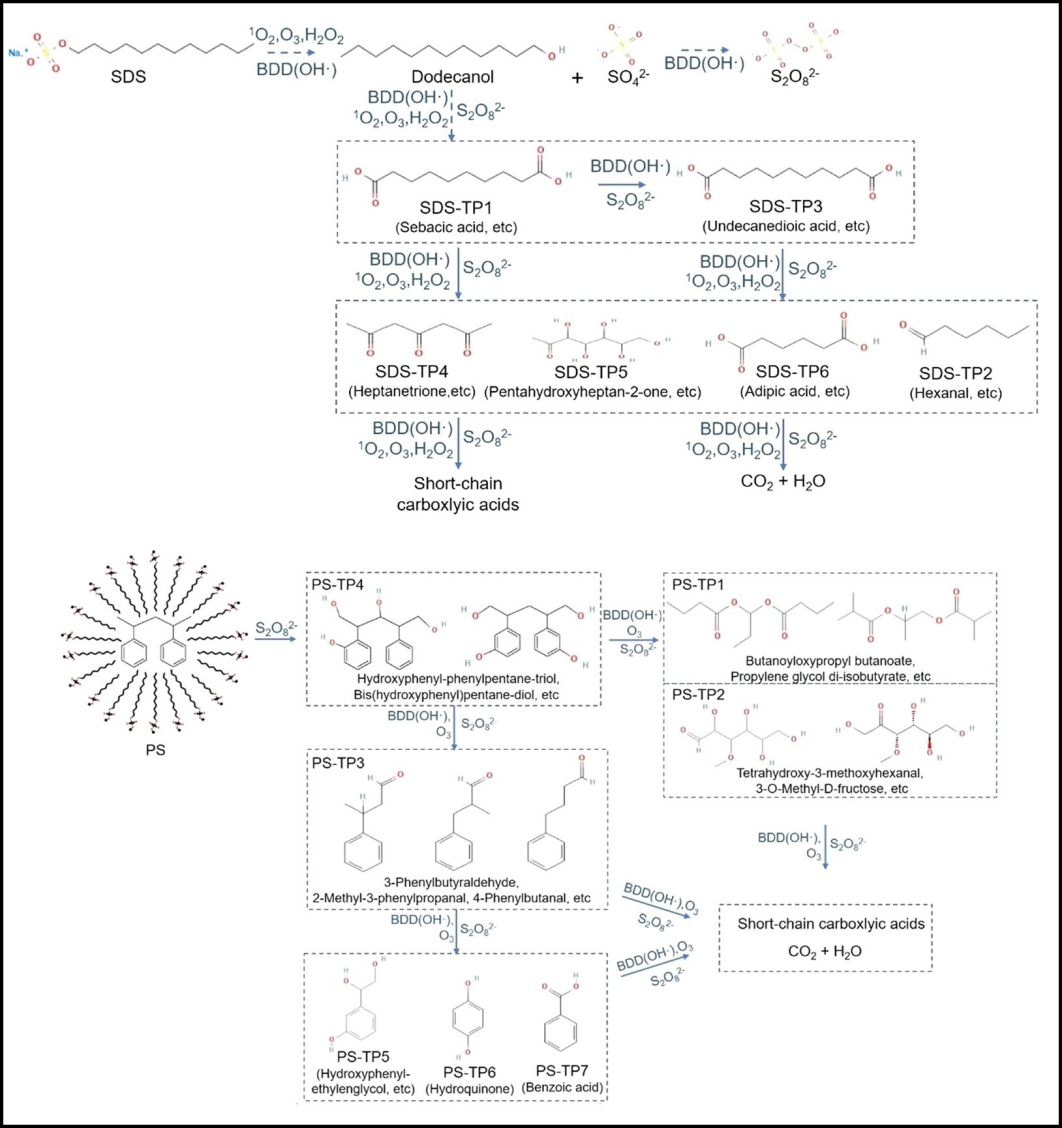
Proposed pathway of the investigated degradation of (a) SDS and (b) PS microplastics by SDS‐assisted BDD. The figure was adapted from Lu et al.^[56]^ with permission from the publisher.

Lu et al. investigated the degradation of PS microplastics using sodium dodecyl sulfate (SDS)‐assisted electrochemical advanced oxidation processes (EAOP).^[56]^ PS is hydrophobic, and the SDS surfactant helps dissolve and mobilize PS within the solution by lowering the surface tension. The electrochemical behavior and decomposition of SDS, PS, and their mixture was studied using boron‐doped diamond (BDD) as anode and platinum as cathode in a 30 mL single‐cell in 0.2 M Na_2_SO_4_ electrolyte solution. SDS showed oxidation peaks at 0.8 to 1.0 V vs. saturated calomel electrode, which increased with concentration, indicating efficient anodic oxidation. PS microplastics alone displayed a low current response due to hydrophobicity, which hindered their reaction with ⋅OH radicals. Adding 1.8 mM SDS improved the current response, lowered the onset potential from 1.32 to 1.25 V, and enhanced ⋅OH radical production, aiding in the oxidation and degradation of PS microplastics. SEM analysis revealed the presence of cracks and cavities on the PS microplastic surface after ECAT reaction, confirming PS degradation. Furthermore, the average size of PS microplastics decreased from 18.5 to 7.9 μm, and the degradation increased from 3.83 % to 42.5 % with SDS‐assisted electrochemical oxidation. The identified decomposition products of SDS led to a proposed pathway involving initial alkyl‐sulfate hydrolysis mediated by electrogenerated ⋅OH radicals at the electrode surface. This process generated several products, such as dibasic acids like SDS‐TP1 (sebacic acid) and SDS‐TP3 (undecane dioic acid), along with other short‐chain alcohols, aldehydes, and carboxylic acids (Figure 16a). During the oxidation of PS microplastics, (Figure 16b) a product with diphenyl rings (PS‐TP4, C_17_H_20_O_4_) was identified. This product could correspond to bis(hydroxyphenyl) pentane‐diol, hydroxyphenyl‐phenylpentane‐triol, or other isomers. Two main subsequent degradation steps were observed for PS‐TP4. PS‐TP3 (C_11_H_20_O_4_) was formed through further alkyl‐cleavage at the phenylethyl position. Following this, 3‐hydroxyphenylethylenglycol (PS‐TP5, C_8_H_10_O_3_), hydroquinone (PS‐TP6, C_6_H_6_O_2_), and benzoic acid (PS‐TP7, C_7_H_6_O_2_) were produced via additional oxidation at the phenylethyl position or the benzene ring. Another pathway identified for PS‐TP3 involved attacking the benzene ring to open it, resulting in the formation of esters, aldehydes, and alcohols (PS‐TP1 and PS‐TP2). These carboxylated and hydroxylated products were consistent with the results detected in FTIR spectra The degradation mechanism of PS microplastics in SDS‐assisted EAOP primarily involves highly oxidizing radicals like persulfate and ⋅OH. SDS enhances the production of persulfate and stable oxidants such as ozone (O_3_), which effectively attack PS molecules, aiding in their dispersion and improving interaction with oxidizing agents. Keeping the SDS concentration below its critical micelle concentration ensures it acts as monomers, preventing hindrance from micelle formation. These findings show how SDS‐assisted electrochemical advanced oxidation enhances the degradation of hydrophobic pollutants by producing reactive species and improving the interactions between plastics and oxidants.

## 5 Photoelectrocatalysis of Plastics

PECAT combines the principles of PCAT and ECAT to drive chemical reactions using both light and electrical energy. This hybrid approach can significantly enhance the efficiency and selectivity of processes aimed at plastic upcycling, transforming waste plastics into valuable chemicals and materials. PECAT systems employ cells that harvest light energy to facilitate chemical reactions through intervening electrical processes. The preparation of photoelectrocatalysts involves synthesizing semiconductor materials, incorporating co‐catalysts, and assembling them onto conductive substrates such as fluorine‐doped tin oxide. A straightforward PECAT system includes UV or visible light‐active semiconductors like TiO_2_, which serves as the photoanode. The photoanode is connected through a potentiostat to the cathode, typically an inert conductor such as Pt, within an electrolyte solution to complete the electrical circuit. Similar to an electrochemical cell, the anode and cathode compartments are usually separated by a semi‐permeable membrane to prevent product crossover, which could reverse the chemical reaction due to opposite polarities. An applied potential is introduced to accelerate the chemical reactions. There have been advances to improve the efficiency of PECAT water splitting to O_2_ and H_2_.^[94]^ However, in a PECAT water‐splitting process, the oxidation half‐reaction results in the O_2_ evolution reaction. Therefore, it is more energy efficient if we can replace O_2_ production with alternative anodic reactions such as removal of organic compounds from wastewater or waste plastics oxidation.[^95^, ^96^]

**Figure 17:**
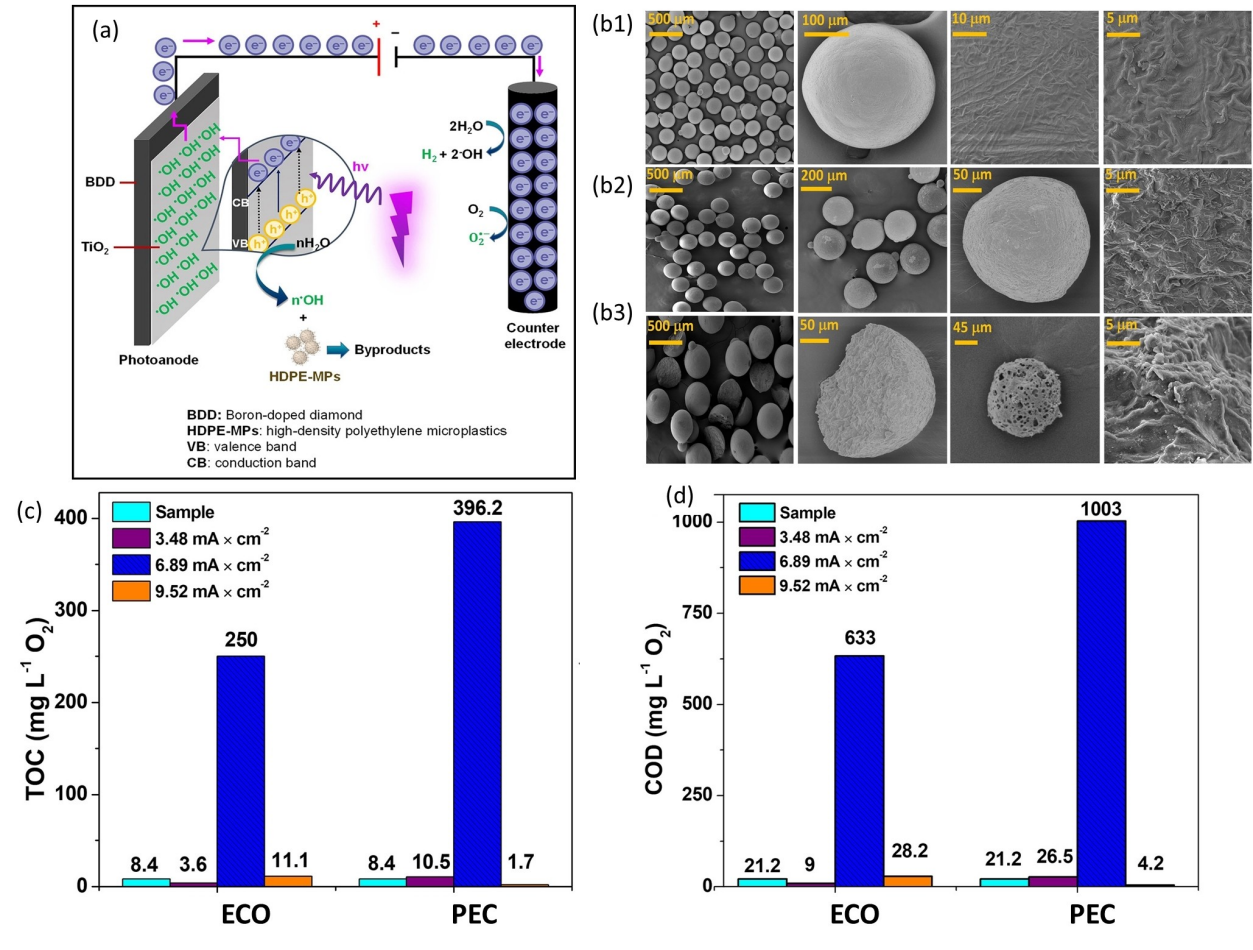
(a) Schematic of photoelectrocatalytic system with TiO_2_/BDD as photoanode and Pt as counter electrode for HDPE microplastic depolymerization. (b) SEM images of HDPE at different scales, (b1) pristine HDPE, (b2) after electrochemical oxidation (EO), (b3) after photoelectrocatalysis (PEC). (c) TOC analysis and (d) COD analysis of the filtered solution of the samples treated at different current densities. These figures were adapted from Quilumbaquin et al.^[97]^ with permission from the publisher.

Quilumbaquin et al. developed a PECAT system using TiO_2_‐modified BDD as the photoanode to decompose HDPE microplastics suspended in 0.1 M Na_2_SO_4_ electrolyte under UV irradiation while simultaneously producing H_2_ at a Pt cathode (Figure 17a).^[97]^ The authors compared the performance of the PECAT approach with ECAT using bare BDD as the anode. SEM analysis revealed changes in HDPE microplastics morphology during both ECAT and PECAT processes. Initially, microplastics were smooth, compact spheres as shown in Figure 17b1; however, the microplastics exhibited amorphous shapes with surface cracks and cavities post ECAT and PECAT treatment (Figure 17b2). During PECAT, microplastics decomposed into hemispheres and fragments with honeycomb‐like porosity (Figure 17b3), indicating interactions with the BDD/TiO_2_ photoanode and UV light. From photo‐ and electro‐catalytic experiments, the photoanode demonstrated higher decomposition efficiency, as evident from total organic carbon (TOC) and chemical oxygen demand (COD) analyses (**Figures** 17c and d). The TOC analysis showed the breakdown of microplastics into different organic compounds, and the COD measured the oxygen required to oxidize these substances, reflecting the extent of decomposition. At a current density of 6.89 mA/cm^2^, the TOC due to the production of organic compounds increased to 250 mg/L for ECAT and 396.2 mg/L for PECAT. The COD results showed 633 mg/L for ECAT and 1003 mg/L for PECAT, indicating the decomposition of the solid plastics into soluble organic compounds such as aldehydes and ketones into the electrolyte. This suggests that PECAT, leveraging the synergistic effects of PCAT and ECAT, offers improved performance and efficiency compared to purely PCAT or ECAT systems, enabling better plastic decomposition and product selectivity control.^[98]^ However, the authors did not report the applied potentials at which both ECAT and PECAT were performed; therefore, it is inconclusive which electrochemical approach is more efficient for plastic decomposition.

## 6 Perspective and Outlook for Improving Plastic Upcycling via Non‐Thermal Routes

### 6.1 Process Development: Introduction of Reactive Functional Groups to the Polymer Backbone

A more controllable approach to chemically recycle or upcycle polyolefins is through the addition of cleavable linkages/functional groups, along the polymer backbone, that allow selective deconstruction. PE and PP are linked by C‐C and C‐H bonds that make them chemically unreactive and highly resistant to decomposition compared to the other plastics containing C‐O and C‐N bonds (e. g., PET, polyurethane) or plastics containing polar groups (e. g., polymethyl methacrylate, PVC). One possible strategy to activate these inert plastics is to introduce reactive functional groups such as alcohols, carbonyls, sulfonates, esters, or amines onto the polymer backbone. This strategy has provided opportunities for further chemical modification to specific applications.

For example, Xing et al. showed relatively higher plastic upcycling efficiency to formic acid of about 16.5 % from PEG and 13.4 % from polyacrylamide (PAM) as compared to 0.44 % from PE.^[91]^ PEG contains polar groups (−OH) and (−O−), which makes it readily soluble in reaction media such as acetonitrile, and PAM easily dissolves in water as it contains a hydrophilic group (−NH_2_). The dissolved plastics can diffuse easily to the surface of a photocatalyst and undergo upcycling reactions. Soo et al. demonstrated >95 % conversion of hydroxyl‐terminated polymers such as PEG, its co‐block polymer with polycaprolactone (PCL‐PEG‐PCL), and PE monoalcohol into formic acid and formate esters.^[99]^ They prepared vanadium (V) complexes supported by hydrazone‐imidate ligands to serve as photocatalysts that absorb visible light through ligand‐to‐metal charge‐transfer processes to selectively cleave the C‐C bonds adjacent to alcohol groups present in the polymer backbone (Table 1).

**Table 1:** Cascade C‐C bond cleavage in macromolecular hydroxyl functionalized biodegradable (entries 1 and 2) and non‐biodegradable (entries 3 and 4) polymers. In all the polymeric substrates, the sites of C‐C bond cleavage are highlighted in red, with multiple, red‐colored C‐C bonds indicating the cascade shortening of the polymer chain. The nuclear magnetic resonance yields of the identified products are shown.

Entry	Macromolecule, μmol	Solvent	Temperature, °C	Time of Reaction, days	Products yield, %
1	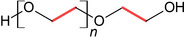	CD_3_CN	rt	2.5	HCOOMe 75±4
	PEG				
2	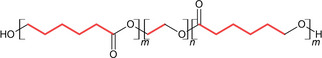	CD_3_CN	rt	2	HCOOH 70±4
	PCL‐PEG‐PCL				
3	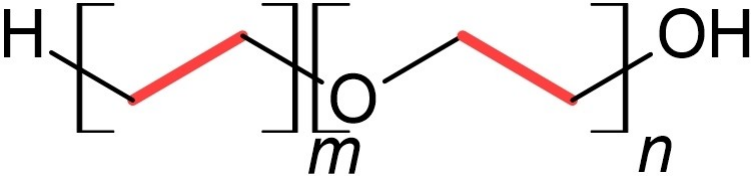	CD_3_CN/ toluene‐d_8_	85	7	HCOOH 6±1
	PE‐PEG				
4	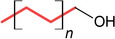	CD_3_CN/ toluene‐d_8_	85	6	HCOOH 5±2
	PE‐monoalcohol				

PET is composed of terephthalate and ethylene groups joined together with ester linkages and represents another example of a functionalized polymer that can successfully be upcycled,[^54^, ^61^, ^62^, ^63^, ^100^] The ester functional group makes PET readily decompose into its monomers under alkaline conditions (1–5 M NaOH or KOH, at 60 °C to 80 °C). Monomer terephthalic acid can be separated by adjusting the pH to <3, and the ethylene glycol monomer can then be further converted into value‐added products such as formic acid via ECAT with Faradaic efficiencies >95 %.[^61^, ^62^] Thus, we surmise that introducing functional groups on inert polymers represents a novel strategy to enable their decomposition and upcycling into valuable products.

Macromolecule→Solvent,Conversion>95%Photocatalyst,airorO2,whiteLEDHCOOH+HCOOMe+Otherproducts

### 6.2 Process Development: Polymer Functionalization Followed by Depolymerization/Upcycling

**Figure 18:**
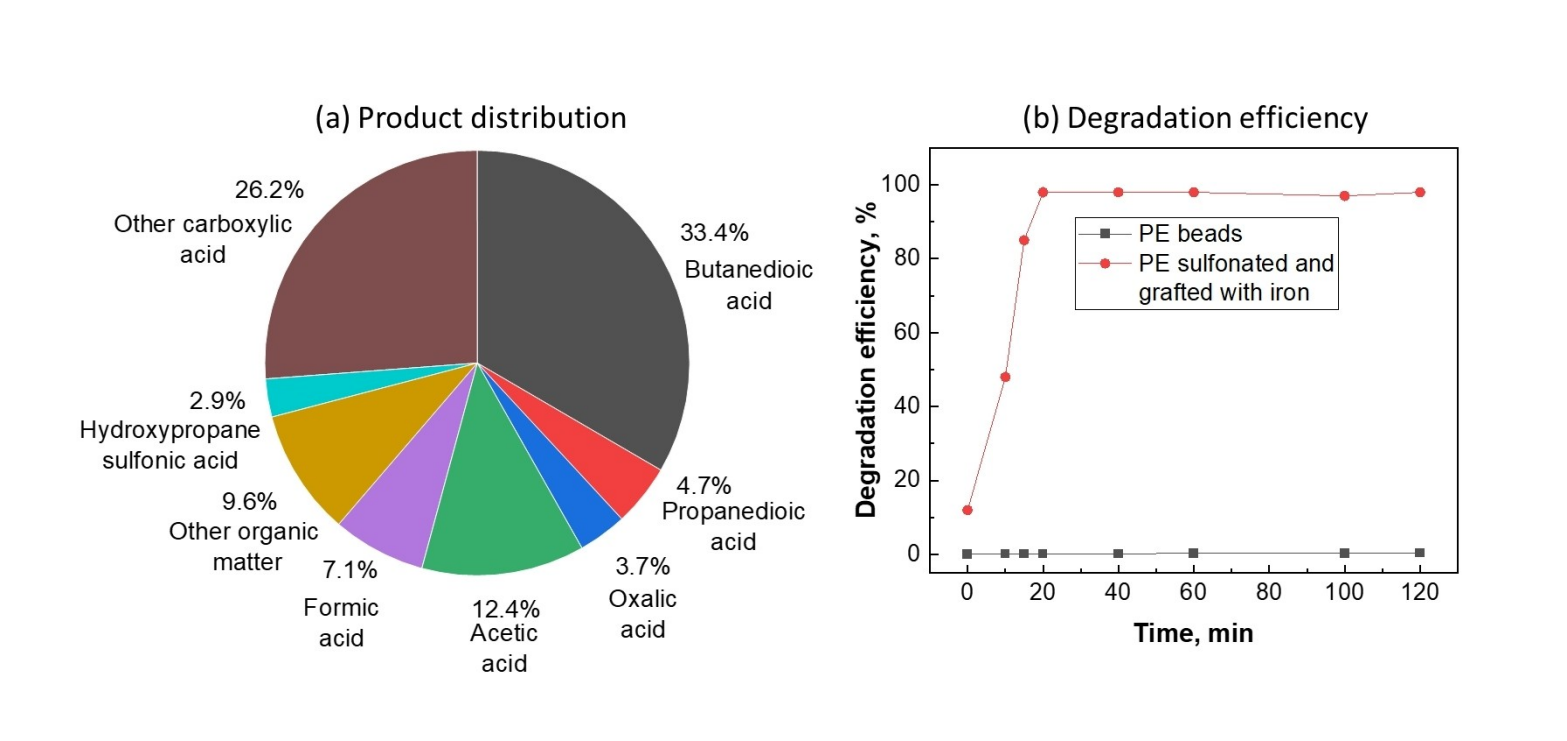
Sulphonated and iron‐grafted PE conversion by Fenton reaction: (a) product distribution, (b) decomposition efficiency. The figures were adapted from Chow et al.^[101]^

Chow et al. demonstrated complete conversion of PE and PP by adopting Fenton technology to convert functionalized polyolefins to organic acids.^[101]^ They demonstrated that PE and PP could not be directly converted via Fenton reaction; however, introducing sulfonate groups followed by Fe^III^ grafting onto the polymer backbone enabled their decomposition.^[101]^ Commercial pellets of LDPE, HDPE, and PP were first grinded into 125 to 250 μm‐sized powders, dissolved in chloroform at 65 °C, and then sulfonated (at every fourth ethylene unit on average) using chlorosulfonic acid. This was followed by grafting of Fe^III^ using FeCl_3_ as the precursor. The treated PE‐SO_3_‐Fe was suspended in water in presence of excess H_2_O_2_ to initiate the Fenton reaction. In less than 20 min, ≈100 % conversion of the sulfonated PE was observed and generated ≈26.7 % CO_2_ and a mixture of carboxylic acids as shown in Figure 18. In this process, excess H_2_O_2_ was used to initiate the Fenton reaction for PE and PP conversion, which resulted in a mixture of carboxylic acids and CO_2_ (Equations (14) to 16.

(14) $${\mathrm{Fe}}^{3+}\;+\;H_{2}O_{2}\to {\mathrm{Fe}}^{2+}\;+\;{{\mathrm{HO}}_{2}}^{.}\;+\;H^{+}\;$$

(15) $${\mathrm{Fe}}^{2+}\;+\;H_{2}O_{2}\;+\;H^{+}\to {\mathrm{Fe}}^{3+}\;+\;H_{2}O\;+\;\cdot \mathrm{OH}\;$$

(16) $$\mathrm{PP}\;\mathrm{or}\;\mathrm{PE}\;+\cdot \mathrm{OH}\to \mathrm{Carboxylic}\;\mathrm{acids}\;+\;{\mathrm{CO}}_{2}\;+\;\mathrm{CO}\;$$

A recent study by Botte′s group introduced an electrochemical method for functionalizing LDPE at low oscillating cell potential of ±1 V in acidic solutions containing transition metal salt (copper sulfate or nickel sulfate).^[102]^ Electrolysis with Cu and Ni electrodes produced new oxygen‐containing groups (C‐O, C‐O, and C‐C) on the polymer surface, with oxygen content increasing to 6.3 % for Cu and 2.7 % for Ni, as confirmed by FTIR and X‐ray photoelectron spectroscopy analysis, respectively. SEM‐energy dispersive X‐ray images of functionalized polymers revealed Cu and Ni nanoparticles, indicating strong metal‐LDPE interactions that likely enhanced oxidation as well as scission of C‐C bonds leading to formation of smaller organic molecules such as dodecanoic acid.

### 6.3 Catalyst Development for Plastic Upcycling via PCAT, ECAT, and PECAT

Catalyst development through innovative material design and advanced catalytic processes can offer sustainable solutions to plastic waste management. Given that relatively few PCAT and ECAT catalysts have been explored for plastic conversion, this discussion highlights some catalysts that have been used to transform other chemical compounds such as biogenic and synthetic polymers, organic compounds present in wastewater, or VOCs. Catalysts effective at converting organic compounds and biogenic polymers can be beneficial for plastic upcycling due to their proficiency in breaking down complex hydrocarbons. These catalysts are designed to facilitate oxidation or reduction reactions, making them versatile for targeting the strong C‐C and C‐H bonds found in plastics. Their ability to selectively cleave specific bonds and generate valuable by‐products under mild conditions is particularly advantageous. Additionally, their efficiency, stability, and selectivity in degrading robust organic materials suggest they can be adapted or optimized for plastic polymers, providing a promising pathway for sustainable conversion of plastic waste into useful products

#### 6.3.1 Photocatalysts for PCAT

Photocatalysts that are effective at converting biomass‐derived organic compounds can also be applied to plastic upcycling due to their ability to break down complex compounds into useful by‐products. Their demonstrated performance in facilitating redox reactions under light irradiation makes them valuable tools for sustainable conversion of plastic waste.

TiO_2_ is one of the most common and effective photocatalysts studied for plastic conversion, as shown in this review. Mixed metal oxides are excellent alternatives, particularly for their activity under visible light. Examples include Bi_2_WO_6_, Bi_2_MoO_6_, Bi_2_O_3_, and BiVO_4_, which have shown notable photocatalytic activity toward the decomposition of organic pollutants and VOCs.[^103^, ^104^, ^105^, ^106^, ^107^] Graphene and its derivatives offer high theoretical specific surface areas (≈2630 m^2^/g),^[108]^ superior carrier mobility [≈20 m^2^/(V ⋅ s)],^[108]^ high adsorption capacity for organic molecules (≈1381 to 2887 mg/g),^[109]^ and good electrical conductivity (2000 S/m),^[108]^ all of which enhance their photocatalytic activity. Earth abundant C_x_N_y_ catalysts (e. g., g‐C_3_N_4,_ C_3_N_3_, C_2_N, C_3_N, and C_3_N_5_) are emerging photocatalysts that can be studied for plastic conversion owing to their unique advantages, including strong visible light absorption (bang gap 1.7–2.7 eV), chemical stability, and suitable oxidation abilities, making them highly prospective for future research and application.^[110]^ Iron‐based photocatalysts such as Fe_2_O_3_, Fe_3_O_4_, FeOOH, BiFeO_3_, ZnFe_2_O_4_, Fe‐based MOF, and others are also suitable candidates owing to their abundance, eco‐friendliness, visible light absorbance with band gap ranging from 2.1–2.7 eV, and exceptional photocatalytic performance.^[111]^ Advanced techniques in photocatalyst development, such as nano structuring (i. e., nanorods,^[112]^ nanowire,^[113]^ and nanoflowers^[114]^) and core‐shell structures,^[115]^ further enhance catalytic performance by maximizing surface area and tuning the active site environment.^[111]^

Further improvements in catalyst development include creating a type II heterojunction, which this paper shows enhances plastic photocatalytic conversion. Type II heterojunction combines two different semiconductor materials with staggered band alignment, improving the separation and migration of photo‐generated charge carriers and enhancing PCAT efficiency. The CB of one semiconductor aligns with the VB of the other semiconductor, forming a staggered energy band structure. The type II heterojunction configuration facilitates the movement of e^−^ to the lower energy CB and h^+^ to the higher energy VB of the adjacent material, mitigating e^−^ and h^+^ recombination. The improved separation of these charge carriers enables the sustained and effective redox reactions, which are critical for breaking down organic pollutants and waste (micro)plastics. Examples include BiVO_4_/TiO_2_, SnO_x_/Zn_2_SnO_4_, CoO/WO_3_, g‐C_3_N_4_/ZnO, SnO_2_/TiO_2_, and others.[^116^, ^117^, ^118^, ^119^]

Another promising alternative is the Z‐scheme system (Figure 12), inspired by natural photosynthesis. This involves two different photocatalyst materials linked by a mediator, such as a solid‐state conductor or a liquid redox couple. The system mimics the “Z” shape of the energy diagram observed in plants’ photosystems I and II. It comprises two photocatalysts with different band gaps, typically a wide‐band gap photocatalyst and a narrow‐band gap photocatalyst. This setup enables high redox potential on both sides to perform oxidation and reduction, leading to more effective degradation and conversion of targeted pollutants. Notable Z‐scheme systems demonstrating excellent photocatalytic activity include WO_3_/g‐C_3_N_4_, C_3_N_4_/TiO_2_, CdS/WO_3_, and others.[^120^, ^121^, ^122^, ^123^]

Doping foreign elements can affect the morphology, particle size, and surface structure of photocatalysts.^[124]^ Proper doping narrows the band gap and creates defect sites like oxygen vacancies, improving photocatalytic activity, while improper doping can cause charge carrier recombination, decreasing efficiency.^[81]^ For example, metal doping (e. g., noble metals, 3D transition metal ions, alkali earth metals, and lanthanides) lowers the band gap energy and improves the catalytic efficiency in the visible‐light region.^[124]^ Additionally, non‐metal doping (e. g., nitrogen, sulfur, carbon) enhances visible‐light absorption by creating intermediate energy levels between the CBs and the VBs of semiconductors. Metal and non‐metal cations doped into wide band gap semiconductors (e. g., TiO_2_, SrTiO_3_, ZnO, and ZnS) efficiently degrade VOCs, improving their photocatalytic performance under visible light.[^124^, ^125^]

Adding noble metals such as Pt, Au, or Ag to photocatalysts like TiO_2_ has been shown to enhance the photocatalytic decomposition of VOCs, including toluene, trichloroethylene, and styrene.[^126^, ^127^] These noble metals act as e^−^ sinks, allowing photogenerated e^−^ to transfer to the surface of the noble metals while photogenerated h^+^ remain on the catalyst surface. This separation decreases e^−^ and h^+^ recombination and enhances photocatalytic activity toward the targeted pollutants.^[128]^

#### 6.3.2 Electrocatalysts for ECAT

Electrocatalysts that are effective for converting biogenic or synthetic organic compounds can also be applied to plastic upcycling, leveraging their proven performance in breaking down complex molecules into valuable by‐products and offering promising potential for sustainable conversion of plastic waste.

Electrocatalysts suitable for PET electrochemical reforming can also treat PE, PP, and PS. Noble metal Pd‐based electrocatalysts could electrochemically reform plastic PET hydrolysate into formate as one of the upcycled products,[^61^, ^66^, ^129^] while non‐noble electrocatalysts catalysts like CuO, Co‐Ni_3_N, NiCo_2_O_4_, and Ni(OH)_2_ are also effective for this process.[^130^, ^131^, ^132^, ^133^] Transition metal phosphides based on Ni, Fe, and Co are cost‐effective alternatives to noble‐metal‐based electrocatalysts with high electrical conductivity.^[134]^ For example, Ni_2_P nanoparticles grown on nickel foam have successfully oxidized 5‐hydroxymethyl furfural (HMF) to 2,5‐furandicarboxylic acid (FDCA).^[135]^ Other electrocatalysts, including CoP, Fe‐CoP, and FeP‐MoO_2_, can oxidize biogenic polymers (e. g., lignin, cellulose) into useful products.[^136^, ^137^] Metal oxides prepared on Ti substrates, such as RuO_2_, IrO_2_, SnO_2_, have shown promise in breaking down complex polymers like lignin under mild electrochemical conditions to produce vanillin and vanillic acid.^[138]^

Noble metals like Pd, Ru, Rh along with transition metals such as Cu, Ni, and Zn on a conductive support such as carbon felt can perform electrocatalytic hydrogenation (ECH) of bio‐oil‐derived oxygenated compounds, converting benzaldehyde to benzyl alcohol,[^139^, ^140^, ^141^] furfural to methyl furan, HMF to dimethyl furan, and so forth.[^140^, ^142^, ^143^] ECH upgrades biomass‐derived compounds into renewable fuels by adding hydrogen or removing oxygen and nitrogen using electricity, thus increasing the energy density by enhancing the hydrogen‐to‐carbon ratio.^[139]^ Additionally, the Pt‐Ru bimetallic electrocatalyst on carbon support functions effectively as an electrochemical hydrodeoxygenation (EC‐HDO) catalyst, converting biomass‐derived oxygenates like phenol into cyclohexane with ≈30 % selectivity.^[144]^ These electrocatalysts are promising for facilitating ECH or EC‐HDO of waste plastics to produce value‐added products. Furthermore, Pt and RuO_2_ can also generate value‐added chemicals such as octane using the anodic electrocatalytic decarboxylation reaction (ECDX) of valeric acid via the Kolbe electrolysis reaction and butene, butanol, and butanoic acid from non‐Kolbe electrolysis.[^59^, ^145^, ^146^] Thus, ECAT represents a novel approach to upcycling plastic‐derived organic compounds via coupled ECH and ECDX reactions.

Electrochemistry offers a promising pathway for converting plastic waste into valuable chemicals. By applying renewable electricity, this process can break down complex polymers into smaller, usable molecules. The resulting products can include fuels, chemical feedstocks, and building blocks for new materials. This method not only addresses the growing issue of plastic waste but also promotes a circular economy by transforming discarded plastics into resources, enhancing sustainability, and decreasing reliance on fossil fuels for chemical production.

#### 6.3.3 Photoelectrocatalysts for PECAT

Photoelectrocatalysts that work well for converting biogenic compounds can also be applied to plastic upcycling due to their strong oxidative properties and the ability to break down complex molecules into valuable by‐products.

TiO_2_ remains a go‐to material for oxidation of organic compounds due to its strong oxidative properties and stability.^[95]^ Reports also show its potential as a photoanode for converting biomass derivatives, such as the conversion of benzyl alcohol to benzaldehyde.^[147]^ Doping TiO_2_ with noble metals like Au, Pd, Ag, or Pt significantly enhances its performance.[^147^, ^148^, ^149^] For instance, undoped TiO_2_ converts benzyl alcohol at 9 % with benzaldehyde selectivity of 60 %. However, doping TiO_2_ with 2 % noble metals improves conversion rates: Pt/TiO_2_ achieves 90 %, Au/TiO_2_ 28 %, Ag/TiO_2_ 18 %, and Pd/TiO_2_ 40 %, all maintaining benzaldehyde selectivity above 97 %.^[150]^ BiVO_4_ and WO_3_ are alternative photoanodes known for their effective visible light absorption.[^151^, ^152^, ^153^] ZnO has also served as an efficient photoelectrocatalysts for the conversion of organic compounds (e. g., norfloxacin, ofloxacin, and toluene),^[154]^ and its activity can be enhanced by doping it with Ni, Co, Ag, or N.^[154]^ Hematite (Fe_2_O_3_) photoanodes are effective in the upcycling of biomass‐derived compounds such as HMF to FDCA, and their performance can be enhanced by doping with Ti or engineering the semiconductor surface with a thin layer of Co‐based cocatalysts, like cobalt phosphate.^[155]^ Adding other cocatalysts such as FeOOH, CoOOH, or NiOOH to Fe_2_O_3_ has demonstrated oxidation of glucose to formate as the main product.^[156]^ Additionally, layered double hydroxides (LDHs) can serve as effective photoelectrocatalysts due to their tunable properties and high surface areas. Examples of LDH photoelectrocatalysts reported for the conversion of organic compounds such as methanol, ethanol, HMF, glucose, furfural, and urea include NiCo, NiFe, NiCoMn, and CoMn.^[157]^

Pursuing PECAT for plastic upcycling harnesses advanced catalysts to convert plastics into valuable products, enhancing sustainability and decreasing waste. The ability of these materials to break down complex molecules through strong oxidative processes presents a significant opportunity for innovative recycling and upcycling methods.

## 7 Summary and Outlook

Polyolefins dominate plastic production but face low recycling rates due to their inertness. This mini review explores various methods for depolymerizing and upcycling inert plastics like PE, PP and PS using non‐thermal techniques such as PCAT and ECAT. While solid‐phase PCAT has been extensively researched, particularly with TiO_2_ nanoparticles embedded in polymer films, liquid‐phase PCAT is a relatively newer area of exploration. However, there is a notable gap in the exploration of ECAT for polyolefin and PS upcycling. The electrocatalytic route presents a novel approach to upcycle waste plastic into value‐added products using renewable electricity, but limited research and development have been undertaken so far, mostly due to the inertness of the PE, PP, and PS for direct electrochemical conversion. However, as this review highlights, conducting pretreatments to include oxygen functionality to the plastics might be the critical step needed to enable their conversion. While these pretreatment approaches have only been demonstrated at the laboratory scale, further research is required to optimize these methods, demonstrate with real mixtures of waste plastics (containing impurities that can affect their conversion), and scale‐up the process.

As summarized in Table 2, the main electrochemical conditions (e. g., photon energy, band gap, applied voltage, conversion, and time on stream) at which these non‐thermal decomposition studies were performed varied for each experiment; therefore, no conclusions can be drawn to determine which electrocatalytic process is the most efficient. Therefore, researchers must try to diligently report and control the main parameters and determine the main performance metrics (e. g., conversion, reaction rate, yield, efficiency, electrode degradation or long‐term stability), which are needed to determine which plastic decomposition method is the most suitable for the different waste plastics. Furthermore, these performance metrics are essential for conducting techno‐economic analysis and life‐cycle assessments necessary to determine how these new non‐thermal technologies compare to traditional thermocatalytic plastic decomposition approaches. Furthermore, noble PCAT, ECAT, and PECAT materials developed for the conversion of biomass‐derived molecules should also be used for the upcycling of synthetic waste polymers. In particular, future research should focus on demonstrating and enhancing the long‐term stability and recyclability, developing environmentally friendly and non‐toxic materials, and exploring the scalability and practical applications of these technologies for waste plastic management.

**Table 2:** Summary of literature review for the decomposition of main waste plastics such as polyethylene (PE), polypropylene (PP), and polystyrene (PS) by solid‐phase photocatalysis, liquid‐phase photocatalysis, electrocatalysis, and photoelectrocatalysis.

Plastic	Catalyst	Photon energy (hν), band gap (eV), or applied potential (V)	Conversion or product formation rate	Key comments	Ref
Solid‐phase photocatalysis					
PP, PE	Anatase TiO_2_	3.6–4.2 eV	NA	Excitation λ_max_ of light absorbing carbonyl groups present in the polymer shifted to a higher wavelength	^[68]^
PP	P‐25 TiO_2_	3.1–3.3 eV	Product formation in μmol/h CO_2_=0.663 CO=0.090	Void formation in the plastic film, decrease in mechanical strength, CO, and CO_2_ generation	^[69]^
PE	P‐25 TiO_2_	4.4 eV	PE decomposition rate 17.8 μg/h	Void formation and decrease in mechanical strength of polymer	^[70]^
PE	P‐25 TiO_2_	3.2 eV	PE decomposition rate 142 μg/h under solar and 283 μg/h under UV	42 % PE weight loss under solar radiation 85 % PE weight loss under UV radiation	^[72]^
PE	CuPc/ TiO_2_	3.2 eV Photovoltage for TiO_2_: 40 μV TiO_2/_CuPc: 70 μV	250 μg/h with CuPc/TiO_2_ and 63 μg/h with TiO_2_	About 40 % PE weight loss from CuPc/TiO_2_ as compared to only 10 % from pristine TiO_2_	^[78]^
PS	CuPc/ TiO_2_	3.1–1.6 eV	6.9 % weight loss in 120 h	Decrease in band gap after CuPc incorporation	^[77]^
PE	TiO_2_ coupled with dye sensitizer malachite green	UV light: 3.9 eV Visible light: 3.1 eV to 1.77 eV	PE decomposition rate 222 μg/day	Decrease in mechanical strength and 50 % PE decomposition in 45 days under visible light	^[79]^
PE	Fe doped ZnO	Sunlight	41.3 % weight loss in 120 h	Decrease in band gap from 3.4 to 3.2 eV on Fe doping	^[80]^
PS	V, Mo, Mn, Cr, W doped TiO2	8 fluorescent bulbs (300 W, UV_B_)	NA	Mo and W doped TiO_2_ showed PS photodegradation better than TiO_2_	^[81]^
PP	TiO_2_‐rGO composite	Direct solar irradiation	NA	Formation of cavities and enhanced PP decomposition compared to pristine TiO_2_ under solar radiation	^[76]^
PE	TiO_2_	3.39 eV	PE decomposition rate 666 μg/h	Radical trapping experiments suggest that photogenerated e^−^ and h^+^ as well O_2_⋅^−^ and ⋅OH took part in the reaction	^[75]^
PE, PP	Polarized KNbO_3_	2.98 eV	CO_2_ generation rate in mg/(g_cat_ ⋅ h) from PE=17 PP=27	Compared to photocatalysts Bi_2_WO_6_, g‐C_3_N_4_, TiO_2_, and WO_3_, P‐KNbO_3_ exhibited 2 to 9 times higher activity for PE conversion	^[82]^
Liquid‐phase photocatalysis					
PE	ZnO	50 W halogen lamp (60–70 klux)	Products formed: ethane, formaldehyde, CO_2_	Increased stiffness, cracks, and chemical changes in LDPE after photocatalysis	^[83]^
PP, PE	Nb_2_O_5_	3.5 eV Lamp intensity 100 mW/cm^2^	Product yield mg/(g_cat_) in 20 h PE: Acetic acid≈0.8 CO_2_ ≈700 PP: Acetic acid≈1.0 CO_2_≈400	100 % photodecomposition of PE and PP to CO_2_ later photoreduced to acetic acid	^[84]^
PE	N doped TiO_2_	Visible light	4–5 % polymer decomposition in 50 h	Plastics of smaller size degraded faster	^[85]^
PS	FeB/TiO_2_	365 nm LED (5 W) lamp	Products include benzaldehyde, phenylacetaldehyde, and benzoic acid	FeB incorporation lowered the activation energy and enhanced e^−^ transfer	^[86]^
PE, PP	Co‐Ga_2_O_3_	Simulated sunlight (AM 1.5 G, 100 mW/cm^2^)	Product formation rate in μmol/(g_cat_ ⋅ h) PE: CO at 158, CO_2_ at 419, H_2_ at 647 PP: CO at 147, CO_2_ at 389, H_2_ at 603	Decrease in band gap, suppression in e^−^ and h^+^ recombination, enhanced charge transfer	^[87]^
PE	Ag_2_O in Fe MOF pores	Ag_2_O: 1.36 eV Fe‐MOF: 2.6 eV	15 % polymer decomposition	PE weight loss for Ag_2_O/Fe‐MOF is 2.2 and 1.7 times higher than Ag_2_O and Fe‐MOF	^[90]^
PE	CdS‐CdO_x_/SiC with Pt 0.5 wt % as cocatalyst	2.1 eV	H_2_ production rate 25 μmol/(g_cat_ ⋅ h)	Photo reforming of PE observed in strong alkaline conditions	^[88]^
PE	Vanadium substituted phosphomolybdic acid/g‐C_3_N_4_ (VPOM/CNNS)	2.95 eV	Formic acid production rate 24.7 μmol/(g_cat_ ⋅ h)	VPOM/CNNS photocatalyst was stable for 100 h.	^[91]^
PE	a. Pt 1 wt % on TiO_2_ b. Pt 1 wt % on C_3_N_4_	100 mW/cm^2^	Products in μmol/(g_cat_ ⋅ h) Ethylene=0.177 Ethane=2.6, Propane=1.45 CO_2_=61.1 H_2_=65.6	Gaseous hydrocarbons yield 1.0 % and CO_2_ 6.1 %	^[55]^
PE	MoS_2_/CdS	CdS: 2.27 eV Decorating CdS with MoS_2_ helped in charge separation	CH_4_ production rate at 196 μmol/(g_cat_ ⋅ h)	Photogenerated h^+^ play crucial role in photocatalytic PE decarboxylation process	^[92]^
PE	Pd ‐TiO_2_	365 nm LED	Products in μmol/(g_cat_ ⋅ h) Propionic acid=265 Ethylene=23.3 Ethane=32.7	Pd helped in more accumulation of charge carriers and suppress e^−^ and h^+^ recombination	^[93]^
Electrocatalysis					
PE	Three working electrodes tested: carbon paper, graphite rod and fluorine doped tin oxide	5.0 V, 130 to 150 C charge passed.	Products in μmol/(cm^2^_cat_ ⋅ h) Ethylene 4.2 to 6 Propylene 0.7 to 1.1	Gaseous hydrocarbons yield 7.6 % and CO_2_ 13.5 %	^[55]^
PS	Boron doped diamond (BDD) anode and assisted using sodium dodecyl sulfate (SDS)	Constant current experiments at 10, 20, 30 mA/cm^2^	10 % to 42.5 % PS decomposition, benzoic acid obtained as one of the products	SDS heled in dissolving PS. Decrease in onset potential after addition of SDS.	^[56]^
Functionalized PE	Ni and Cu electrodes	Switch polarity electrolysis. Cell potential ±1 V	Reaction mass efficiency for product dodecanoic acid is 0.013–0.027 %	Electrochemical functionalization of PE helped in activating it and leading to its depolymerization	^[102]^
PE	Boron doped diamond (BDD)	Constant current experiments at 1.5, 6.9, 9.5 mA/cm^2^	17–55 % polymer decomposition	Aldehydes and ketones were detected as products	^[97]^
Photoelectrocatalysis					
PE	TiO_2_ modified BDD	Band gap 3.32 eV Constant current experiments at 1.5, 6.9, 9.5 mA/cm^2^	24–90 % polymer decomposition	Aldehydes and ketones were detected as products	^[97]^

In summary, despite the environmental and sustainable benefits of the combined PECAT methods for waste plastic decomposition, these technologies remain largely unexplored. Thus, additional research as well as strict testing and reporting methodologies are needed to understand their advantages and disadvantages compared to traditional methods and to identify key areas of research beyond those outlined in this review.

## Conflict of Interests

There are no conflicts to declare.

## Biographical Information

Bhanupriya Boruah pursued her Ph.D. at the Indian Institute of Science (2016 – 2021), focusing on photocatalysis and photoelectrocatalysis for environmental remediation. Currently, she is a Chemical Engineer at the Pacific Northwest National Laboratory (PNNL), where her research focuses on electrocatalysis for wastewater treatment, biomass conversion, and plastic upcycling. Before joining PNNL, Dr. Boruah served as an Archer Daniels Midland (ADM) postdoctoral researcher at the University of Kansas, where she worked on thermocatalytic biomass upgrading.

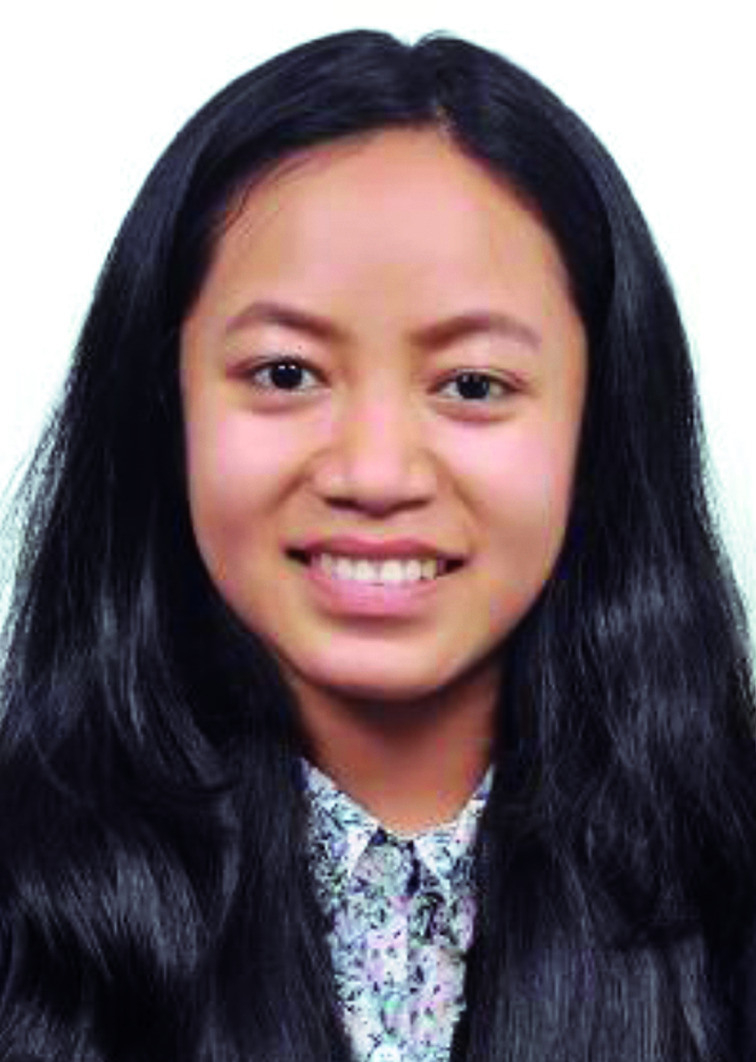

## Biographical Information

Born and raised in Tarragona, Catalonia, Spain, Dr. Juan A. Lopez‐Ruiz earned his bachelor′s degree in Chemical Engineering from Universitat Rovira i Virgili in 2007 and studied at Bucknell University in 2006. He obtained his master′s in 2009 and completed his Ph.D. at the University of Virginia in 2014. Joining the Pacific Northwest National Laboratory in 2015 as a post‐doctoral researcher, he became a full staff member in 2017. Dr. Lopez‐Ruiz focuses on developing electro‐ and thermo‐catalytic processes to generate chemicals and hydrogen from renewable and waste resources as well as seawater.